# Supplementary figures and images for: Assessing the ecological patterns of Aedes aegypti in areas with high arboviral risks in the large city of Abidjan, Côte d’Ivoire
Source: PLoS Negl Trop Dis. 2024 Nov 18;18(11):e0012647. doi: 10.1371/journal.pntd.0012647 (PMC11611265; doi:10.1371/journal.pntd.0012647)

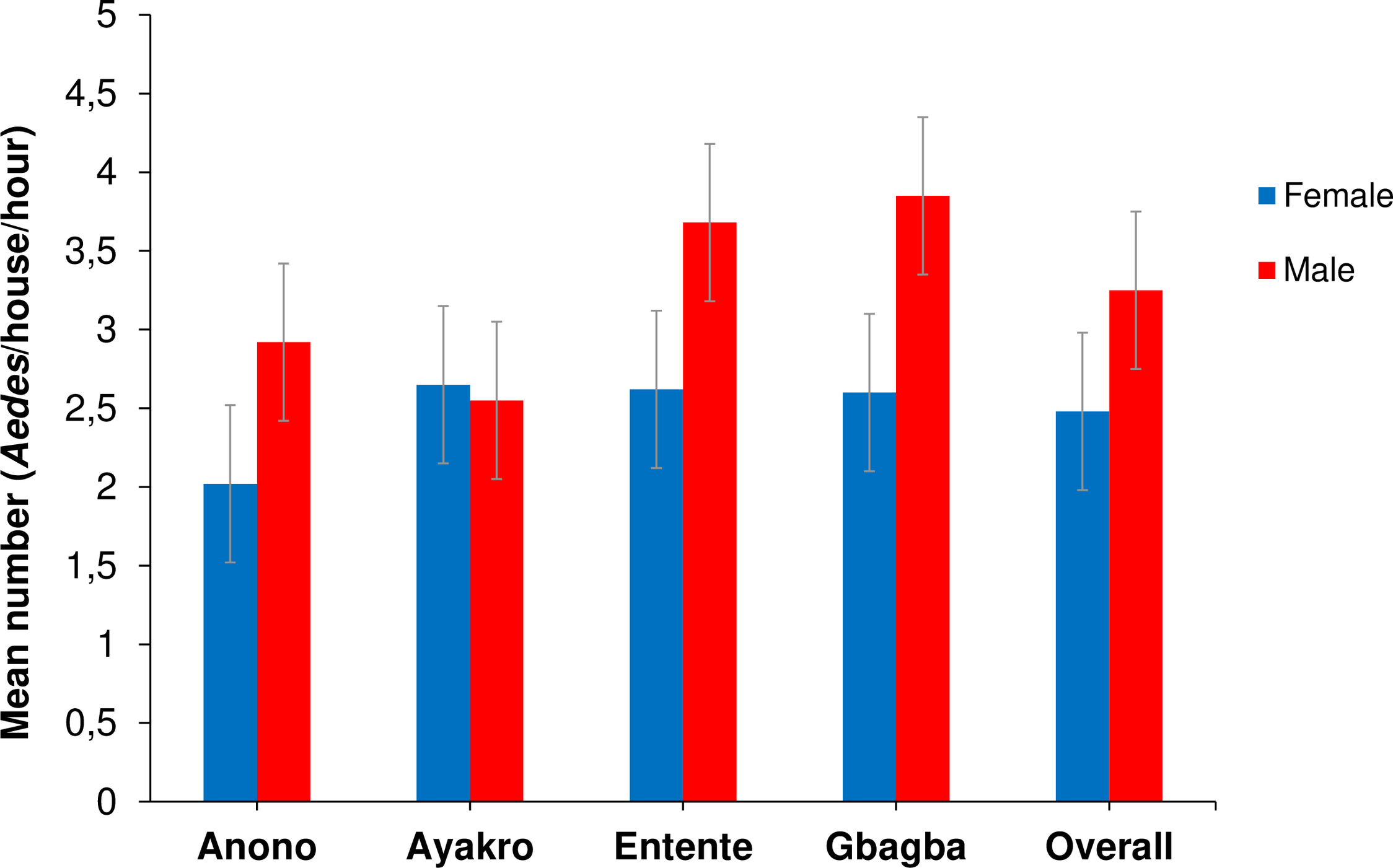

Supplement: S1 Fig — Error bars show the standard error (SE). (TIF) [file pntd.0012647.s001.tif]

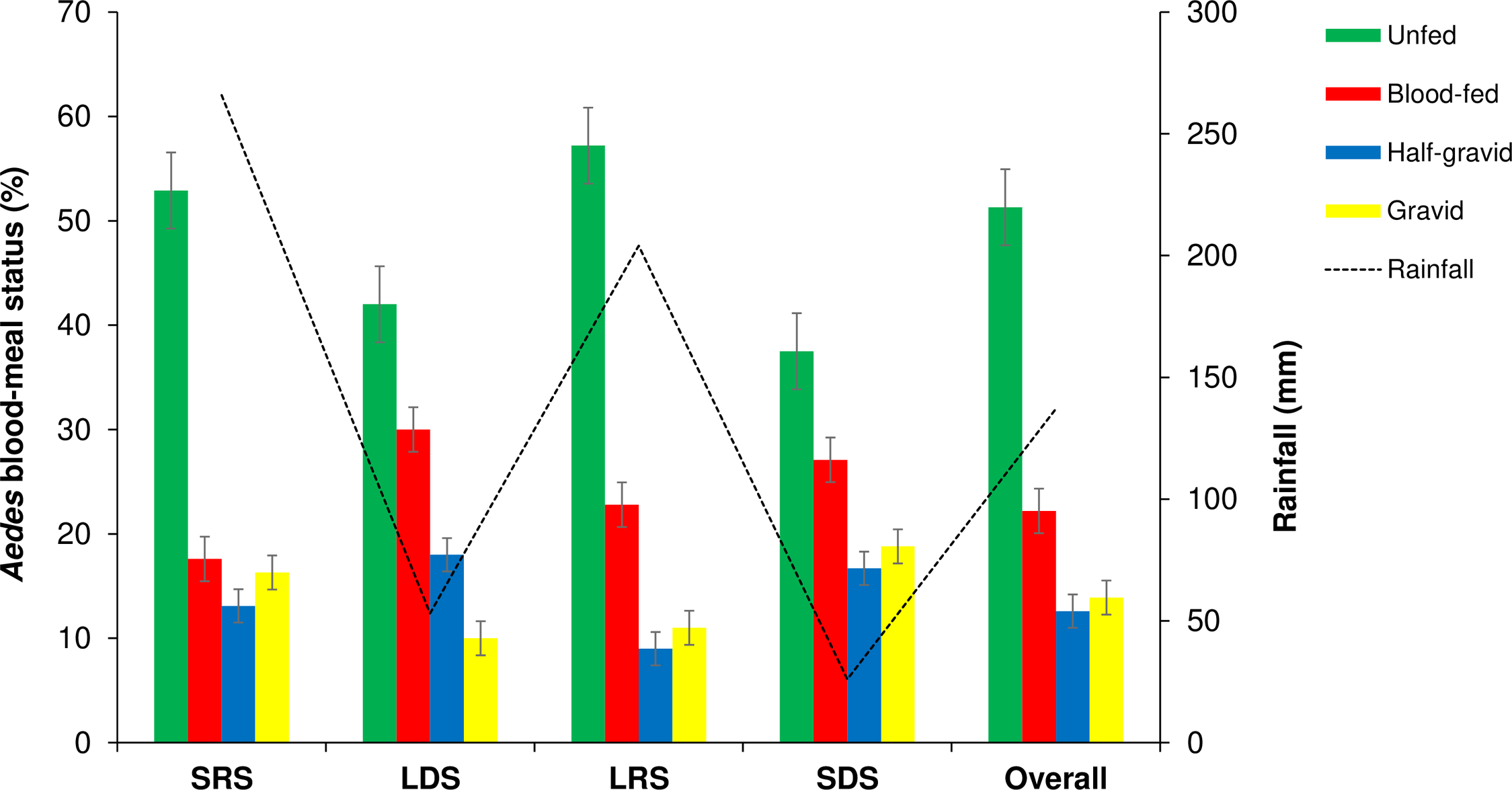

Supplement: S2 Fig — SRS: short rainy season, LDS: long dry season, LRS: long rainy season, SDS: short dry season. Error bars show the standard error (SE). (TIF) [file pntd.0012647.s002.tif]

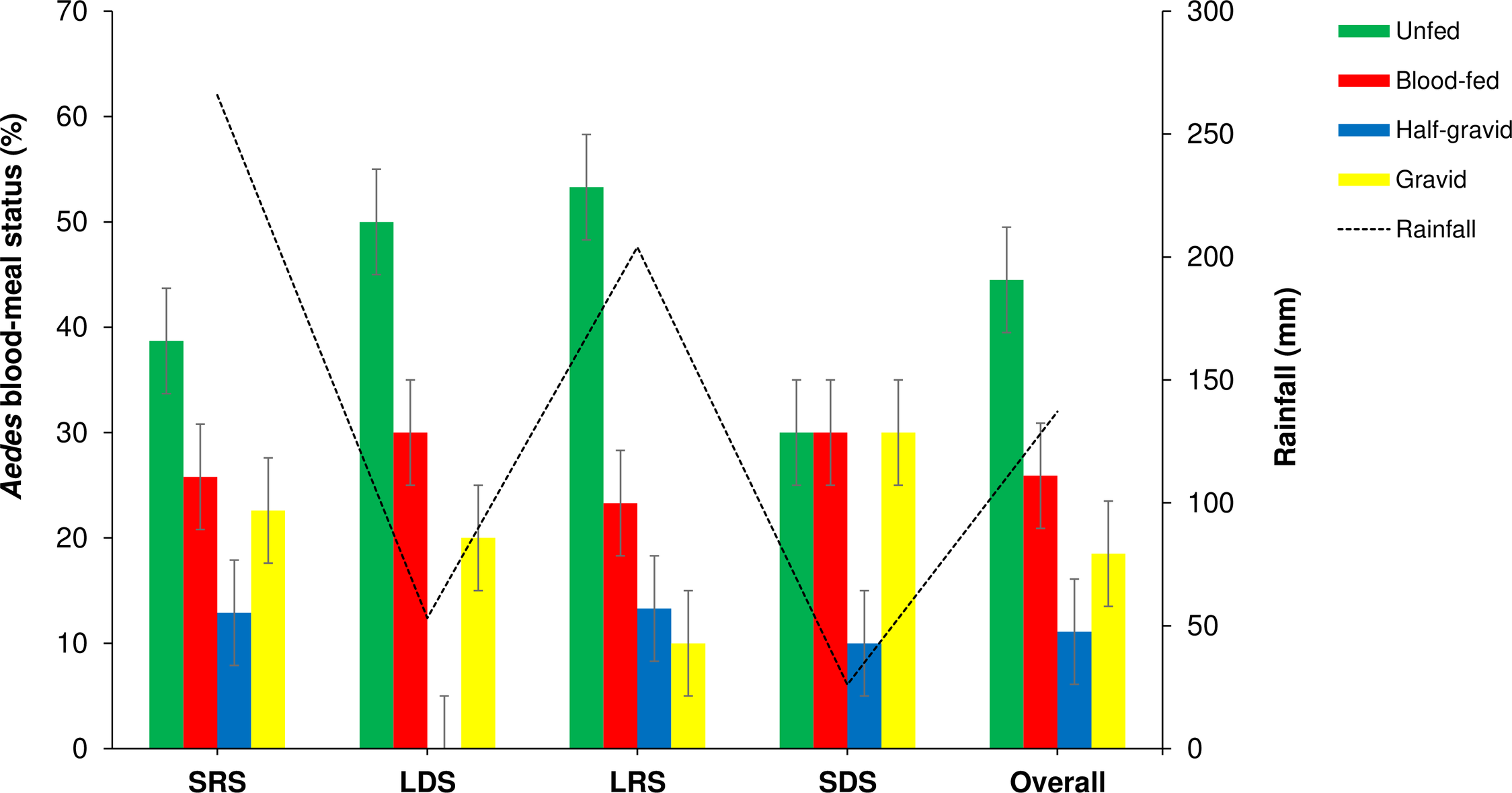

Supplement: S3 Fig — SRS: short rainy season, LDS: long dry season, LRS: long rainy season, SDS: short dry season. Error bars show the standard error (SE). (TIF) [file pntd.0012647.s003.tif]

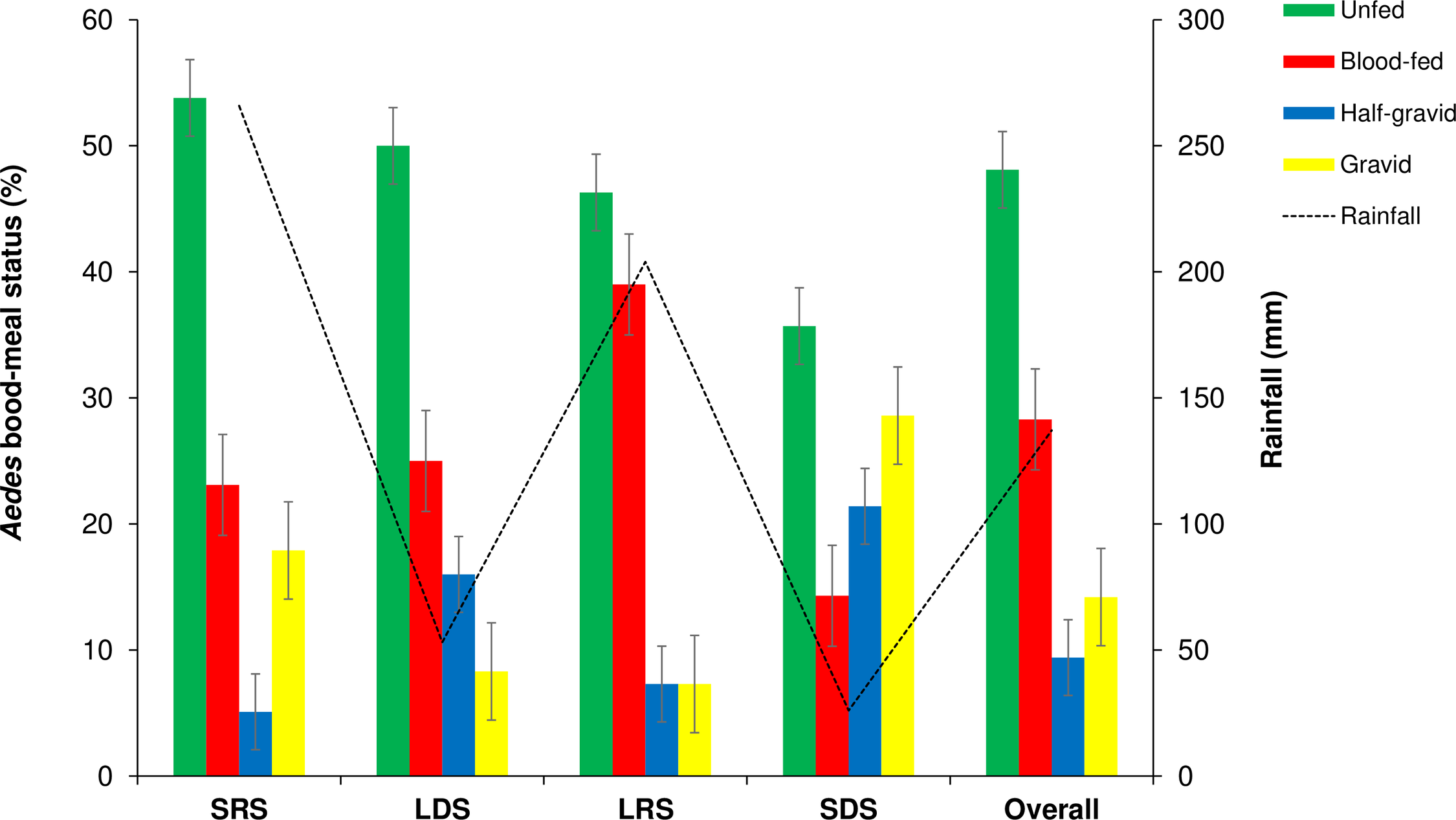

Supplement: S4 Fig — SRS: short rainy season, LDS: long dry season, LRS: long rainy season, SDS: short dry season. Error bars show the standard error (SE). (TIF) [file pntd.0012647.s004.tif]

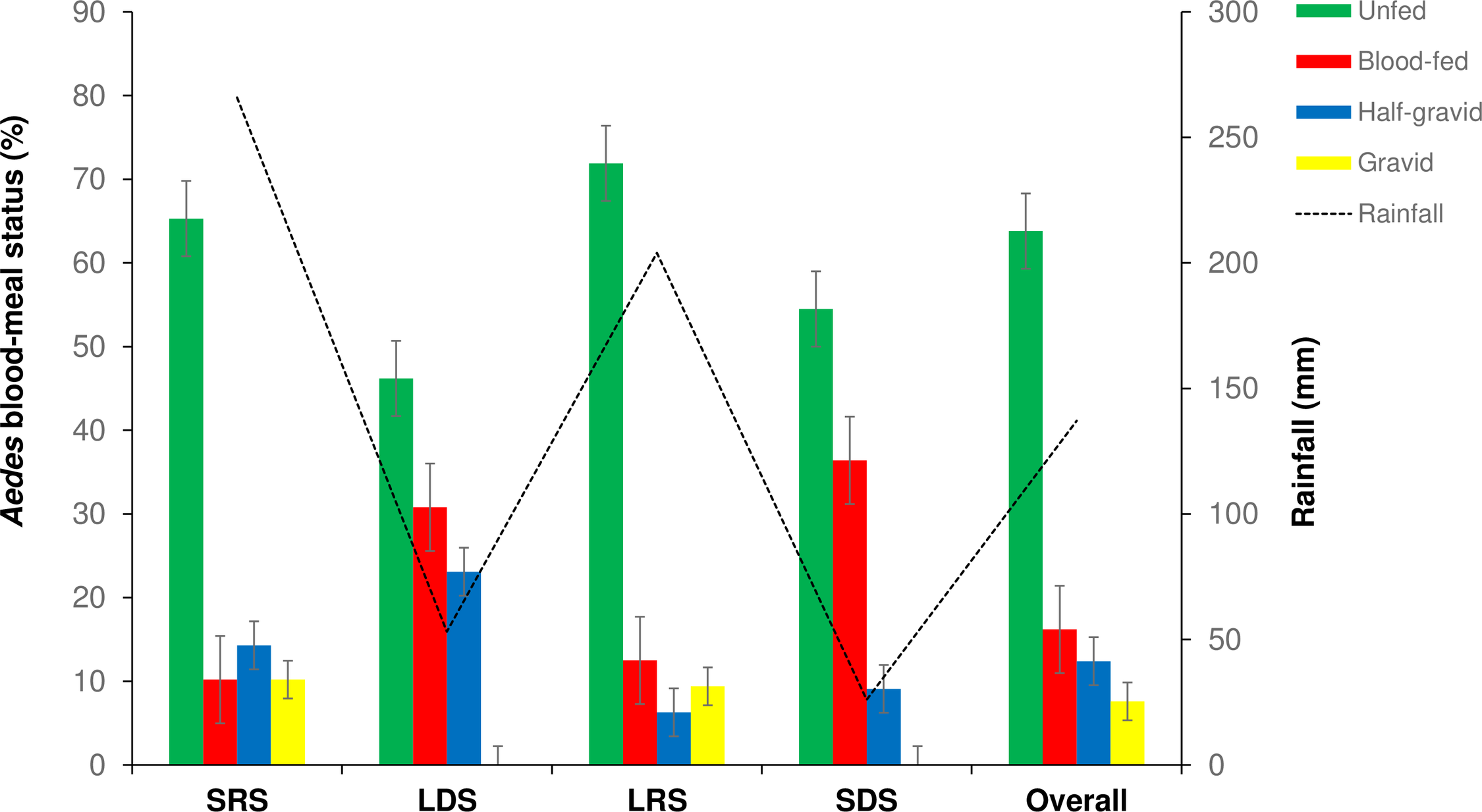

Supplement: S5 Fig — SRS: short rainy season, LDS: long dry season, LRS: long rainy season, SDS: short dry season. Error bars show the standard error (SE). (TIF) [file pntd.0012647.s005.tif]

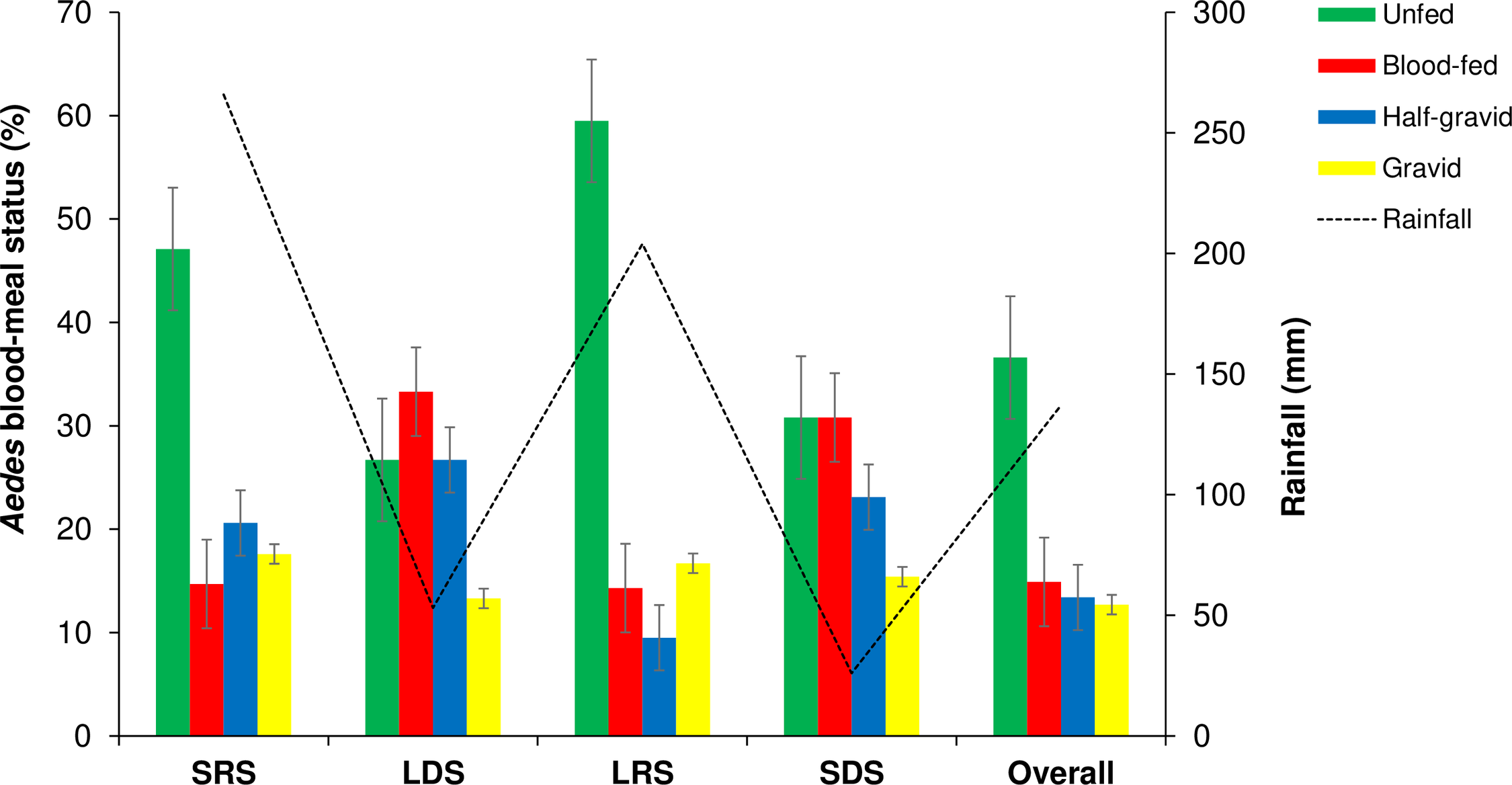

Supplement: S6 Fig — SRS: short rainy season, LDS: long dry season, LRS: long rainy season, SDS: short dry season. Error bars show the standard error (SE). (TIF) [file pntd.0012647.s006.tif]

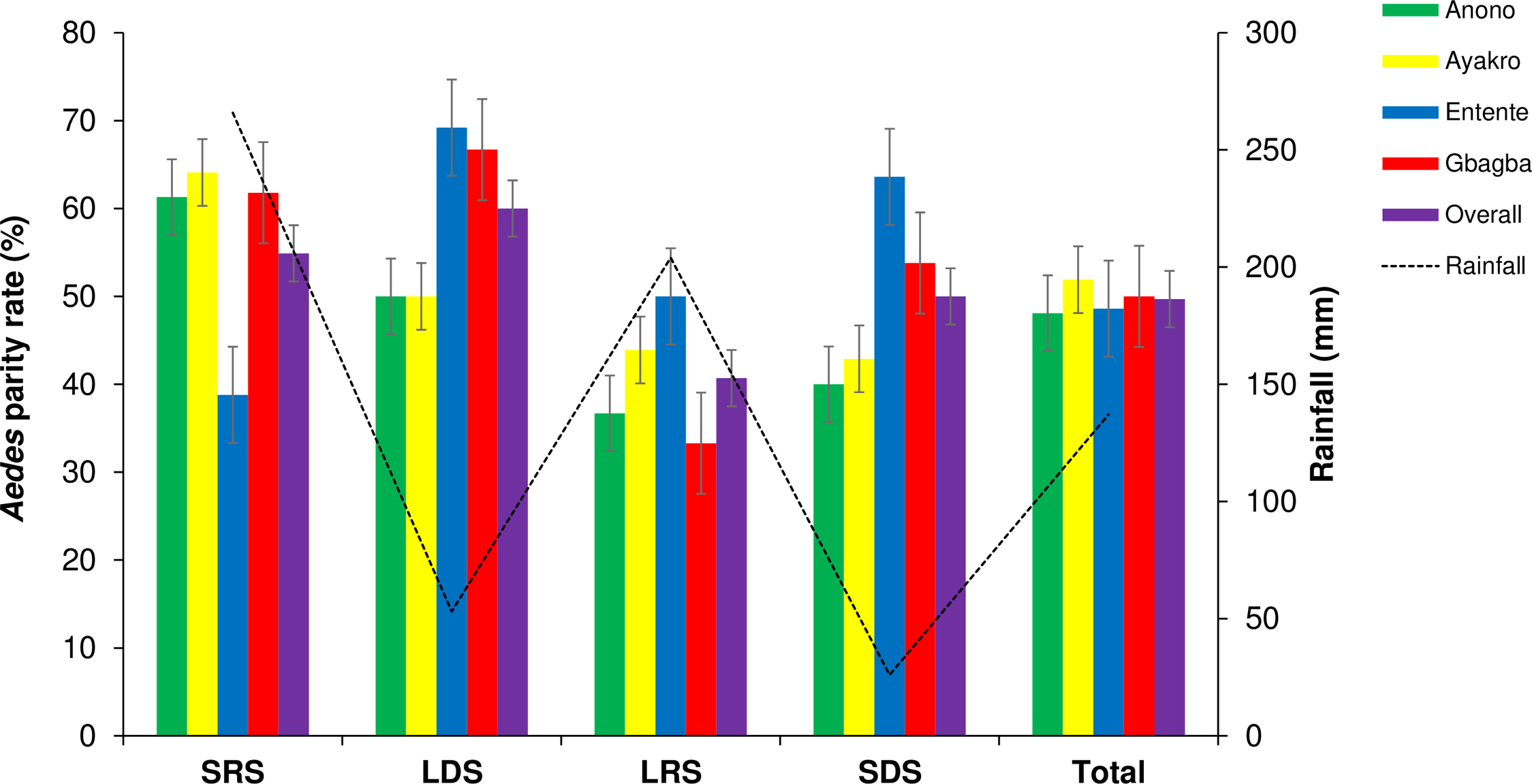

Supplement: S7 Fig — SRS: short rainy season, LDS: long dry season, LRS: long rainy season, SDS: short dry season. Error bars show the standard error (SE). (TIF) [file pntd.0012647.s007.tif]
