# Supplementary material for: Assessing the ecological patterns of Aedes aegypti in areas with high arboviral risks in the large city of Abidjan, Côte d’Ivoire
Source: PLoS Negl Trop Dis. 2024 Nov 18;18(11):e0012647. doi: 10.1371/journal.pntd.0012647 (PMC11611265; doi:10.1371/journal.pntd.0012647)
Supplement: S1 Table — WSC: Water storage containers, DC: Discarded containers, NBS: Natural breeding sites, N: number of wet containers inspected, n: Aedes-positive containers, PW: Percentage of Aedes-positive breeding sites among wet containers, PP: Proportion of each Aedes-positive breeding site type among the all Aedes-positive breeding site types. PW and PP are expressed as a percentage (%), na: not applicable, SRS: short rainy season, LDS: long dry season, LRS: long rainy season, SDS: short dry season. Others is the category of breeding containers composed of hole of brick, shoes, tarp, flower pot, wooden box, mortar, sheet metal. Natural breeding site is composed of water on land, leaf axils, snail shell, tree hole. (DOCX) [file pntd.0012647.s008.docx]

| **S1 Table. Seasonal variations of the abundance of the larval breeding sites of *Aedes aegypti* in domestic and peridomestic premises in the study sites within the city of Abidjan, Côte d’Ivoire from August 2019 to July 2020.** | | | | | | | | | | | | | | | | | | | | | | |
| --- | --- | --- | --- | --- | --- | --- | --- | --- | --- | --- | --- | --- | --- | --- | --- | --- | --- | --- | --- | --- | --- | --- |
| **Study site** | **Premise** | **Breeding**  **site** | **SRS** | | | | **LDS** | | | | **LRS** | | | | **SDS** | | | | **Total** | | | |
|  |  |  | **N** | **n** | **PW** | **PP** | **N** | **n** | **PW** | **PP** | **N** | **n** | **PW** | **PP** | **N** | **n** | **PW** | **PP** | **N** | **n** | **PW** | **PP** |
| **Anono** | Domestic | WSC | 314 | 58 | 18.5 | 63.0 | 127 | 14 | 11.0 | 60.9 | 225 | 28 | 12.4 | 66.7 | 100 | 6 | 6.0 | 85.7 | 766 | 106 | 13.8 | 64.6 |
|  |  | Tires | 64 | 19 | 29.7 | 20.7 | 9 | 3 | 33.3 | 13.0 | 45 | 9 | 20.0 | 21.4 | 3 | 1 | 33.3 | 14.3 | 121 | 32 | 26.4 | 19.5 |
|  |  | DC | 19 | 11 | 57.9 | 12.0 | 6 | 3 | 50.0 | 13.0 | 11 | 4 | 36.4 | 9.5 | 0 | 0 | na | 0.0 | 36 | 18 | 50.0 | 11.0 |
|  |  | NBS | 0 | 0 | na | 0.0 | 0 | 0 | na | 0.0 | 0 | 0 | na | 0.0 | 0 | 0 | na | 0.0 | 0 | 0 | na | 0.0 |
|  |  | Others | 11 | 4 | 36.4 | 4.3 | 7 | 3 | 42.9 | 13.0 | 7 | 1 | 14.3 | 2.4 | 1 | 0 | 0.0 | 0.0 | 26 | 8 | 30.8 | 4.9 |
|  |  | **Total** | **408** | **92** | **22.5** | **100** | **149** | **23** | **15.4** | **100** | **288** | **42** | **14.6** | **100** | **104** | **7** | **6.7** | **100** | **949** | **164** | **17.3** | **100** |
|  | Peridomestic | WSC | 1 | 0 | 0.0 | 0.0 | 1 | 0 | 0.0 | 0.0 | 1 | 1 | 100 | 1.6 | 0 | 0 | na | 0 | 3 | 1 | 33.3 | 0.5 |
|  |  | Tires | 229 | 52 | 22.7 | 83.9 | 138 | 42 | 30.4 | 97.7 | 148 | 50 | 33.8 | 78.1 | 70 | 25 | 35.7 | 100 | 585 | 169 | 28.9 | 87.1 |
|  |  | DC | 23 | 7 | 30.4 | 11.3 | 0 | 0 | na | 0.0 | 22 | 10 | 45.5 | 15.6 | 1 | 0 | 0.0 | 0 | 46 | 17 | 37.0 | 8.8 |
|  |  | NBS | 0 | 0 | na | 0.0 | 0 | 0 | na | 0.0 | 0 | 0 | na | 0.0 | 0 | 0 | na | 0 | 0 | 0 | na | 0.0 |
|  |  | Others | 6 | 3 | 50.0 | 4.8 | 1 | 1 | 100 | 2.3 | 9 | 3 | 33.3 | 4.7 | 0 | 0 | na | 0 | 16 | 7 | 43.8 | 3.6 |
|  |  | **Total** | **259** | **62** | **23.9** | **100** | **140** | **43** | **30.7** | **100** | **180** | **64** | **35.6** | **100** | **71** | **25** | **35.2** | **100** | **650** | **194** | **29.8** | **100** |
|  | Total | WSC | 315 | 58 | 18.4 | 37.7 | 128 | 14 | 10.9 | 21.2 | 226 | 29 | 12.8 | 27.4 | 100 | 6 | 6.0 | 18.75 | 769 | 107 | 13.9 | 29.9 |
|  |  | Tires | 293 | 71 | 24.2 | 46.1 | 147 | 45 | 30.6 | 68.2 | 193 | 59 | 30.6 | 55.7 | 73 | 26 | 35.6 | 81.25 | 706 | 201 | 28.5 | 56.1 |
|  |  | DC | 42 | 18 | 42.9 | 11.7 | 6 | 3 | 50.0 | 4.5 | 33 | 14 | 42.4 | 13.2 | 1 | 0 | 0.0 | 0 | 82 | 35 | 42.7 | 9.8 |
|  |  | NBS | 0 | 0 | na | 0.0 | 0 | 0 | na | 0.0 | 0 | 0 | na | 0.0 | 0 | 0 | na | 0 | 0 | 0 | na | 0.0 |
|  |  | Others | 17 | 7 | 41.2 | 4.5 | 8 | 4 | 50.0 | 6.1 | 16 | 4 | 25.0 | 3.8 | 1 | 0 | 0.0 | 0 | 42 | 15 | 35.7 | 4.2 |
|  |  | **Total** | **667** | **154** | **23.1** | **100** | **289** | **66** | **22.8** | **100** | **468** | **106** | **22.6** | **100** | **175** | **32** | **18.3** | **100** | **1599** | **358** | **22.4** | **100** |
| **Ayakro** | Domestic | WSC | 384 | 89 | 23.2 | 80.2 | 362 | 42 | 11.6 | 79.2 | 330 | 66 | 20.0 | 79.5 | 180 | 31 | 17.22 | 93.9 | 1256 | 228 | 18.2 | 81.4 |
|  |  | Tires | 9 | 7 | 77.8 | 6.3 | 10 | 3 | 30.0 | 5.7 | 21 | 8 | 38.1 | 9.6 | 3 | 1 | 33.33 | 3.0 | 43 | 19 | 44.2 | 6.79 |
|  |  | DC | 12 | 3 | 25.0 | 2.7 | 9 | 4 | 44.4 | 7.5 | 22 | 9 | 40.9 | 10.8 | 2 | 0 | 0 | 0.0 | 45 | 16 | 35.6 | 5.71 |
|  |  | NBS | 1 | 0 | 0.0 | 0.0 | 0 | 0 | na | 0.0 | 0 | 0 | 0.0 | na | 0 | 0 | na | 0.0 | 1 | 0 | 0.0 | 0.00 |
|  |  | Others | 25 | 12 | 48.0 | 10.8 | 9 | 4 | 44.4 | 7.5 | 5 | 0 | 0.0 | 0.0 | 2 | 1 | 50 | 3.0 | 41 | 17 | 41.5 | 6.07 |
|  |  | **Total** | **431** | **111** | **25.8** | **100** | **390** | **53** | **13.6** | **100** | **378** | **83** | **22.0** | **100** | **187** | **33** | **17.65** | **100** | **1386** | **280** | **20.2** | **100** |
|  | Peridomestic | WSC | 0 | 0 | na | 0.0 | 4 | 1 | 25.0 | 20 | 1 | 0 | 0.0 | 0.0 | 0 | 0 | na | 0.0 | 5 | 1 | 20.0 | 1.2 |
|  |  | Tires | 49 | 25 | 51.0 | 71.4 | 18 | 4 | 22.2 | 80 | 38 | 23 | 60.5 | 65.7 | 22 | 7 | 31.8 | 77.8 | 127 | 59 | 46.5 | 70.2 |
|  |  | DC | 26 | 9 | 34.6 | 25.7 | 3 | 0 | 0.0 | 0 | 24 | 12 | 50.0 | 34.3 | 3 | 2 | 66.7 | 22.2 | 56 | 23 | 41.1 | 27.4 |
|  |  | NBS | 0 | 0 | na | 0.0 | 0 | 0 | na | 0 | 0 | 0 | na | 0.0 | 0 | 0 | na | 0.0 | 0 | 0 | na | 0.0 |
|  |  | Others | 2 | 1 | 50.0 | 2.9 | 0 | 0 | na | 0 | 0 | 0 | na | 0.0 | 0 | 0 | na | 0.0 | 2 | 1 | 50.0 | 1.2 |
|  |  | **Total** | **77** | **35** | **45.5** | **100** | **25** | **5** | **20.0** | **100** | **63** | **35** | **55.6** | **100** | **25** | **9** | **36.0** | **100** | **190** | **84** | **44.2** | **100** |
|  | Total | WSC | 384 | 89 | 23.2 | 61.0 | 366 | 43 | 11.7 | 74.1 | 331 | 66 | 19.9 | 55.9 | 180 | 31 | 17.2 | 73.8 | 1261 | 229 | 18.2 | 62.9 |
|  |  | Tires | 58 | 32 | 55.2 | 21.9 | 28 | 7 | 25.0 | 12.1 | 59 | 31 | 52.5 | 26.3 | 25 | 8 | 32.0 | 19.0 | 170 | 78 | 45.9 | 21.4 |
|  |  | DC | 38 | 12 | 31.6 | 8.2 | 12 | 4 | 33.3 | 6.9 | 46 | 21 | 45.7 | 17.8 | 5 | 2 | 40.0 | 4.8 | 101 | 39 | 38.6 | 10.7 |
|  |  | NBS | 1 | 0 | 0.0 | 0.0 | 0 | 0 | na | 0.0 | 0 | 0 | na | 0.0 | 0 | 0 | na | 0.0 | 1 | 0 | 0.0 | 0.0 |
|  |  | Others | 27 | 13 | 48.1 | 8.9 | 9 | 4 | 44.4 | 6.9 | 5 | 0 | 0.0 | 0.0 | 2 | 1 | 50.0 | 2.4 | 43 | 18 | 41.9 | 4.9 |
|  |  | **Total** | **508** | **146** | **28.7** | **100** | **415** | **58** | **14.0** | **100** | **441** | **118** | **26.8** | **100** | **212** | **42** | **19.8** | **100** | **1576** | **364** | **23.1** | **100** |
| **Entente** | Domestic | WSC | 330 | 43 | 13.0 | 59.7 | 184 | 19 | 10.3 | 90.5 | 236 | 35 | 14.8 | 63.6 | 135 | 9 | 6.7 | 81.8 | 885 | 106 | 12.0 | 66.7 |
|  |  | Tires | 3 | 2 | 66.7 | 2.8 | 0 | 0 | na | 0.0 | 10 | 0 | 0.0 | 0.0 | 14 | 0 | 0.0 | 0.0 | 27 | 2 | 7.4 | 1.3 |
|  |  | DC | 48 | 18 | 37.5 | 25.0 | 10 | 1 | 10.0 | 4.8 | 25 | 14 | 56.0 | 25.5 | 5 | 0 | 0.0 | 0.0 | 88 | 33 | 37.5 | 20.8 |
|  |  | NBS | 4 | 1 | 25.0 | 1.4 | 3 | 0 | 0.0 | 0.0 | 1 | 0 | 0.0 | 0.0 | 1 | 1 | 100 | 9.1 | 9 | 2 | 22.2 | 1.3 |
|  |  | Others | 13 | 8 | 61.5 | 11.1 | 3 | 1 | 33.3 | 4.8 | 9 | 6 | 66.7 | 10.9 | 7 | 1 | 14.3 | 9.1 | 32 | 16 | 50.0 | 10.1 |
|  |  | **Total** | **398** | **72** | **18.1** | **100** | **200** | **21** | **10.5** | **100** | **281** | **55** | **19.6** | **100** | **162** | **11** | **6.8** | **100** | **1041** | **159** | **15.3** | **100** |
|  | Peridomestic | WSC | 0 | 0 | na | 0.0 | 0 | 0 | na | 0 | 0 | 0 | na | 0.0 | 1 | 0 | 0.0 | 0 | 1 | 0 | 0.0 | 0.0 |
|  |  | Tires | 60 | 9 | 15.0 | 75.0 | 4 | 2 | 50.0 | 100 | 62 | 16 | 25.8 | 84.2 | 13 | 2 | 15.4 | 100 | 139 | 29 | 20.9 | 82.9 |
|  |  | DC | 27 | 3 | 11.1 | 25.0 | 4 | 0 | 0.0 | 0.0 | 3 | 2 | 66.7 | 10.5 | 1 | 0 | 0.0 | 0.0 | 35 | 5 | 14.3 | 14.3 |
|  |  | NBS | 0 | 0 | na | 0.0 | 0 | 0 | na | 0.0 | 0 | 0 | na | 0.0 | 0 | 0 | na | 0.0 | 0 | 0 | na | 0.0 |
|  |  | Others | 0 | 0 | na | 0.0 | 1 | 0 | 0.0 | 0.0 | 3 | 1 | 33.3 | 5.3 | 0 | 0 | na | 0.0 | 4 | 1 | 25.0 | 2.9 |
|  |  | **Total** | **87** | **12** | **13.8** | **100** | **9** | **2** | **22.2** | **100** | **68** | **19** | **27.9** | **100** | **15** | **2** | **13.3** | **100** | **179** | **35** | **19.6** | **100** |
|  | Total | WSC | 330 | 43 | 13.0 | 51.2 | 184 | 19 | 10.3 | 82.6 | 236 | 35 | 14.8 | 47.3 | 136 | 9 | 6.6 | 69.2 | 886 | 106 | 12.0 | 54.6 |
|  |  | Tires | 63 | 11 | 17.5 | 13.1 | 4 | 2 | 50.0 | 8.7 | 72 | 16 | 22.2 | 21.6 | 27 | 2 | 7.4 | 15.4 | 166 | 31 | 18.7 | 16.0 |
|  |  | DC | 75 | 21 | 28.0 | 25.0 | 14 | 1 | 7.1 | 4.3 | 28 | 16 | 57.1 | 21.6 | 6 | 0 | 0.0 | 0.0 | 123 | 38 | 30.9 | 19.6 |
|  |  | NBS | 4 | 1 | 25.0 | 1.2 | 3 | 0 | 0.0 | 0.0 | 1 | 0 | 0.0 | 0.0 | 1 | 1 | 100 | 7.7 | 9 | 2 | 22.2 | 1.0 |
|  |  | Others | 13 | 8 | 61.5 | 9.5 | 4 | 1 | 25.0 | 4.3 | 12 | 7 | 58.3 | 9.5 | 7 | 1 | 14.3 | 7.7 | 36 | 17 | 47.2 | 8.8 |
|  |  | **Total** | **485** | **84** | **17.3** | **100** | **209** | **23** | **11.0** | **100** | **349** | **74** | **21.2** | **100** | **177** | **13** | **7.3** | **100** | **1220** | **194** | **15.9** | **100** |
| **Gbagba** | Domestic | WSC | 321 | 55 | 17.1 | 55 | 302 | 27 | 8.9 | 55.1 | 372 | 66 | 17.7 | 53.2 | 147 | 17 | 11.6 | 70.8 | 1142 | 165 | 14.4 | 55.6 |
|  |  | Tires | 29 | 14 | 48.3 | 14 | 44 | 12 | 27.3 | 24.5 | 66 | 17 | 25.8 | 13.7 | 10 | 2 | 20.0 | 8.3 | 149 | 45 | 30.2 | 15.2 |
|  |  | DC | 37 | 22 | 59.5 | 22 | 14 | 8 | 57.1 | 16.3 | 36 | 33 | 91.7 | 26.6 | 14 | 5 | 35.7 | 20.8 | 101 | 68 | 67.3 | 22.9 |
|  |  | NBS | 1 | 0 | 0.0 | 0 | 2 | 0 | 0.0 | 0.0 | 1 | 0 | 0.0 | 0.0 | 0 | 0 | na | 0.0 | 4 | 0 | 0.0 | 0.0 |
|  |  | Others | 14 | 9 | 64.3 | 9 | 7 | 2 | 28.6 | 4.1 | 20 | 8 | 40.0 | 6.5 | 3 | 0 | 0.0 | 0.0 | 44 | 19 | 43.2 | 6.4 |
|  |  | **Total** | **402** | **100** | **24.9** | **100** | **369** | **49** | **13.3** | **100** | **495** | **124** | **25.1** | **100** | **174** | **24** | **13.8** | **100** | **1440** | **297** | **20.6** | **100** |
|  | Peridomestic | WSC | 0 | 0 | na | 0.0 | 0 | 0 | na | 0.0 | 1 | 1 | 100 | 1.6 | 4 | 2 | 50.0 | 20 | 5 | 3 | 60.0 | 2.7 |
|  |  | Tires | 44 | 15 | 34.1 | 53.6 | 15 | 8 | 53.3 | 72.7 | 37 | 17 | 45.9 | 27.4 | 6 | 5 | 83.3 | 50 | 102 | 45 | 44.1 | 40.5 |
|  |  | DC | 34 | 11 | 32.4 | 39.3 | 9 | 3 | 33.3 | 27.3 | 49 | 23 | 46.9 | 37.1 | 8 | 1 | 12.5 | 10 | 100 | 38 | 38.0 | 34.2 |
|  |  | NBS | 1 | 0 | 0.0 | 0.0 | 0 | 0 | na | 0.0 | 8 | 2 | 25.0 | 3.2 | 5 | 0 | 0.0 | 0 | 14 | 2 | 14.3 | 1.8 |
|  |  | Others | 2 | 2 | 100 | 7.1 | 5 | 0 | 0.0 | 0.0 | 31 | 19 | 61.3 | 30.6 | 50 | 2 | 4.0 | 20 | 88 | 23 | 26.1 | 20.7 |
|  |  | **Total** | **81** | **28** | **34.6** | **100** | **29** | **11** | **37.9** | **100** | **126** | **62** | **49.2** | **100** | **73** | **10** | **13.7** | **100** | **309** | **111** | **35.9** | **100** |
|  | Total | WSC | 321 | 55 | 17.1 | 43.0 | 302 | 27 | 8.9 | 45.0 | 373 | 67 | 18.0 | 36.0 | 151 | 19 | 12.6 | 55.9 | 1147 | 168 | 14.6 | 41.2 |
|  |  | Tires | 73 | 29 | 39.7 | 22.7 | 59 | 20 | 33.9 | 33.3 | 103 | 34 | 33.0 | 18.3 | 16 | 7 | 43.8 | 20.6 | 251 | 90 | 35.9 | 22.1 |
|  |  | DC | 71 | 33 | 46.5 | 25.8 | 23 | 11 | 47.8 | 18.3 | 85 | 56 | 65.9 | 30.1 | 22 | 6 | 27.3 | 17.6 | 201 | 106 | 52.7 | 26.0 |
|  |  | NBS | 2 | 0 | 0.0 | 0.0 | 2 | 0 | 0.0 | 0.0 | 9 | 2 | 22.2 | 1.1 | 5 | 0 | 0.0 | 0.0 | 18 | 2 | 11.1 | 0.5 |
|  |  | Others | 16 | 11 | 68.8 | 8.6 | 12 | 2 | 16.7 | 3.3 | 51 | 27 | 52.9 | 14.5 | 53 | 2 | 3.8 | 5.9 | 132 | 42 | 31.8 | 10.3 |
|  |  | **Total** | **483** | **128** | **26.5** | **100** | **398** | **60** | **15.1** | **100** | **621** | **186** | **30.0** | **100** | **247** | **34** | **13.8** | **100** | **1749** | **408** | **23.3** | **100** |
| **Overall** | Domestic | WSC | 1349 | 245 | 18.2 | 65.3 | 975 | 102 | 10.5 | 69.9 | 1163 | 195 | 16.8 | 64.1 | 562 | 63 | 11.2 | 84.0 | 4049 | 605 | 14.9 | 67.2 |
|  |  | Tires | 105 | 42 | 40.0 | 11.2 | 63 | 18 | 28.6 | 12.3 | 142 | 34 | 23.9 | 11.2 | 30 | 4 | 13.3 | 5.3 | 340 | 98 | 28.8 | 10.9 |
|  |  | DC | 116 | 54 | 46.6 | 14.4 | 39 | 16 | 41.0 | 11.0 | 94 | 60 | 63.8 | 19.7 | 21 | 5 | 23.8 | 6.7 | 270 | 135 | 50.0 | 15.0 |
|  |  | NBS | 6 | 1 | 16.7 | 0.3 | 5 | 0 | 0.0 | 0.0 | 2 | 0 | 0.0 | 0.0 | 1 | 1 | 100 | 1.3 | 14 | 2 | 14.3 | 0.2 |
|  |  | Others | 63 | 33 | 52.4 | 8.8 | 26 | 10 | 38.5 | 6.8 | 41 | 15 | 36.6 | 4.9 | 13 | 2 | 15.4 | 2.7 | 143 | 60 | 42.0 | 6.7 |
|  |  | **Total** | **1639** | **375** | **22.9** | **100** | **1108** | **146** | **13.2** | **100** | **1442** | **304** | **21.1** | **100** | **627** | **75** | **12.0** | **100** | **4816** | **900** | **18.7** | **100** |
|  | Peridomestic | WSC | 1 | 0 | 0.0 | 0.0 | 5 | 1 | 20.0 | 1.6 | 3 | 2 | 66.7 | 1.1 | 5 | 2 | 40.0 | 4.3 | 14 | 5 | 35.7 | 1.2 |
|  |  | Tires | 382 | 101 | 26.4 | 73.7 | 175 | 56 | 32.0 | 91.8 | 285 | 106 | 37.2 | 58.9 | 111 | 39 | 35.1 | 84.8 | 953 | 302 | 31.7 | 71.2 |
|  |  | DC | 110 | 30 | 27.3 | 21.9 | 16 | 3 | 18.8 | 4.9 | 98 | 47 | 48.0 | 26.1 | 13 | 3 | 23.1 | 6.5 | 237 | 83 | 35.0 | 19.6 |
|  |  | NBS | 1 | 0 | 0.0 | 0.0 | 0 | 0 | 0.0 | 0.0 | 8 | 2 | 25.0 | 1.1 | 5 | 0 | 0.0 | 0.0 | 14 | 2 | 14.3 | 0.5 |
|  |  | Others | 10 | 6 | 60.0 | 4.4 | 7 | 1 | 14.3 | 1.6 | 43 | 23 | 53.5 | 12.8 | 50 | 2 | 4.0 | 4.3 | 110 | 32 | 29.1 | 7.5 |
|  |  | **Total** | **504** | **137** | **27.2** | **100** | **203** | **61** | **30.0** | **100** | **437** | **180** | **41.2** | **100** | **184** | **46** | **25.0** | **100** | **1328** | **424** | **31.9** | **100** |
|  | Total | WSC | 1350 | 245 | 18.1 | 47.9 | 980 | 103 | 10.5 | 49.8 | 1166 | 197 | 16.9 | 40.7 | 567 | 65 | 11.5 | 53.7 | 4063 | 610 | 15.0 | 46.1 |
|  |  | Tires | 487 | 143 | 29.4 | 27.9 | 238 | 74 | 31.1 | 35.7 | 427 | 140 | 32.8 | 28.9 | 141 | 43 | 30.5 | 35.5 | 1293 | 400 | 30.9 | 30.2 |
|  |  | DC | 226 | 84 | 37.2 | 16.4 | 55 | 19 | 34.5 | 9.2 | 192 | 107 | 55.7 | 22.1 | 34 | 8 | 23.5 | 6.6 | 507 | 218 | 43.0 | 16.5 |
|  |  | NBS | 7 | 1 | 14.3 | 0.2 | 5 | 0 | 0.0 | 0.0 | 10 | 2 | 20.0 | 0.4 | 6 | 1 | 16.7 | 0.8 | 28 | 4 | 14.3 | 0.3 |
|  |  | Others | 73 | 39 | 53.4 | 7.6 | 33 | 11 | 33.3 | 5.3 | 84 | 38 | 45.2 | 7.9 | 63 | 4 | 6.3 | 3.3 | 253 | 92 | 36.4 | 6.9 |
|  |  | **Total** | **2143** | **512** | **23.9** | **100** | **1311** | **207** | **15.8** | **100** | **1879** | **484** | **25.8** | **100** | **811** | **121** | **14.9** | **100** | **6144** | **1324** | **21.5** | **100** |
| WSC: Water storage containers, DC: Discarded containers, NBS: Natural breeding sites, N: number of wet containers inspected, n: *Aedes-*positive containers, PW: Percentage of *Aedes*-positive breeding sites among wet containers, PP: Proportion of each *Aedes*-positive breeding site type among the all Aedes-positive breeding site types. PW and PP are expressed as a percentage (%), na: not applicable, SRS: short rainy season, LDS: long dry season, LRS: long rainy season, SDS: short dry season. Others is the category of breeding containers composed of hole of brick, shoes, tarp, flower pot, wooden box, mortar, sheet metal. Natural breeding site is composed of water on land, leaf axils, snail shell, tree hole. | | | | | | | | | | | | | | | | | | | | | | |
