# Supplementary material for: Assessing the ecological patterns of Aedes aegypti in areas with high arboviral risks in the large city of Abidjan, Côte d’Ivoire
Source: PLoS Negl Trop Dis. 2024 Nov 18;18(11):e0012647. doi: 10.1371/journal.pntd.0012647 (PMC11611265; doi:10.1371/journal.pntd.0012647)
Supplement: S3 Table — %: Percentage, n: Number of collected adult Aedes aegypti mosquitoes. (DOCX) [file pntd.0012647.s010.docx]

| **S3 Table. Seasonal variations of *Aedes* *aegypti* adults collected outdoors and indoors of houses of the study sites within the city of Abidjan, Côte d’Ivoire from August 2019 to July 2020.** | | | | | | | | | | | |
| --- | --- | --- | --- | --- | --- | --- | --- | --- | --- | --- | --- |
| **Study site** | **House** | **SRS** | | **LDS** | | **LRS** | | **SDS** | | **Total** | |
|  |  | **n** | **%** | **n** | **%** | **n** | **%** | **n** | **%** | **n** | **%** |
| **Anono** | Outdoors | 83 | 96.5 | 19 | 100 | 72 | 94.7 | 15 | 88.2 | 189 | 95.5 |
|  | Indoors | 3 | 3.5 | 0 | 0 | 4 | 5.3 | 2 | 11.8 | 9 | 4.5 |
|  | **Total** | **86** | **100** | **19** | **100** | **76** | **100** | **17** | **100** | **198** | **100** |
| **Ayakro** | Outdoors | 71 | 97.3 | 23 | 95.8 | 59 | 78.7 | 35 | 97.2 | 188 | 90.4 |
|  | Indoors | 2 | 2.7 | 1 | 4.2 | 16 | 21.3 | 1 | 2.8 | 20 | 9.6 |
|  | **Total** | **73** | **100** | **24** | **100** | **75** | **100** | **36** | **100** | **208** | **100** |
| **Entente** | Outdoors | 116 | 94.3 | 25 | 100 | 67 | 90.5 | 29 | 96.7 | 237 | 94.0 |
|  | Indoors | 7 | 5.7 | 0 | 0 | 7 | 9.5 | 1 | 3.3 | 15 | 6.0 |
|  | **Total** | **123** | **100** | **25** | **100** | **74** | **100** | **30** | **100** | **252** | **100** |
| **Gbagba** | Outdoors | 94 | 92.2 | 30 | 88.2 | 91 | 96.8 | 27 | 96.4 | 242 | 93.8 |
|  | Indoors | 8 | 7.8 | 4 | 11.8 | 3 | 3.2 | 1 | 3.6 | 16 | 6.2 |
|  | **Total** | **102** | **100** | **34** | **100** | **94** | **100** | **28** | **100** | **258** | **100** |
| **Overall** | Outdoors | 364 | 94.8 | 97 | 95.1 | 289 | 90.6 | 106 | 95.5 | 856 | 93.4 |
|  | Indoors | 20 | 5.2 | 5 | 4.9 | 30 | 9.4 | 5 | 4.5 | 60 | 6.6 |
|  | **Total** | **384** | **100** | **102** | **100** | **319** | **100** | **111** | **100** | **916** | **100** |
| %: Percentage, n: Number of collected adult *Aedes aegypti* mosquitoes, SRS: short rainy season, LDS: long dry season, LRS: long rainy season, SDS: short dry season. | | | | | | | | | | | |
