# Supplementary material for: Assessing the ecological patterns of Aedes aegypti in areas with high arboviral risks in the large city of Abidjan, Côte d’Ivoire
Source: PLoS Negl Trop Dis. 2024 Nov 18;18(11):e0012647. doi: 10.1371/journal.pntd.0012647 (PMC11611265; doi:10.1371/journal.pntd.0012647)
Supplement: S4 Table — %: percentage, n: number of Aedes aegypti mosquitoes, SE: standard error, SRS: short rainy season, LDS: long dry season, LRS: long rainy season, SDS: short dry season. (DOCX) [file pntd.0012647.s011.docx]

| **S4 Table. Seasonal variations of the blood-meal status of *Aedes aegypti* females in the study sites within the city of Abidjan, Côte d’Ivoire from August 2019 to July 2020.** | | | | | | | | | | | |
| --- | --- | --- | --- | --- | --- | --- | --- | --- | --- | --- | --- |
| **Study site** | **Blood-meal status** | **SRS** | | **LDS** | | **LRS** | | **SDS** | | **Total** | |
|  |  | **n** | **%** ± SE | **n** | **%** ± SE | **n** | **%** ± SE | **n** | **%** ± SE | **n** | **%** ± SE |
| **Anono** | Unfed | 12 | 38.7 ± 8.7 | 5 | 50.0 ± 15.8 | 16 | 53.3 ± 9.1 | 3 | 30.0 ± 14.5 | 36 | 44.4 ± 5.5 |
|  | Blood fed | 8 | 25.8 ± 7.8 | 3 | 30.0 ± 14.5 | 7 | 23.3 ± 7.7 | 3 | 30.0 ± 14.5 | 21 | 25.9 ± 4.9 |
|  | Half-gravid | 4 | 12.9 ± 6.0 | 0 | 0.0 ± 0.0 | 4 | 13.3 ± 6.2 | 1 | 10.0 ± 9.5 | 9 | 11.1 ± 3.5 |
|  | Gravid | 7 | 22.6 ± 7.5 | 2 | 20.0 ± 12.6 | 3 | 10.0 ± 5.5 | 3 | 30.0 ± 14.5 | 15 | 18.5 ± 4.3 |
|  | **Total** | **31** | **100.0 ± 0.0** | **10** | **100.0 ± 0.0** | **30** | **100.0 ± 0.0** | **10** | **100.0 ± 0.0** | **81** | **100.0 ± 0.0** |
| **Ayakro** | Unfed | 21 | 53.8 ± 8.0 | 6 | 50.0 ± 14.4 | 19 | 46.3 ± 7.8 | 5 | 35.7 ± 12.8 | 51 | 48.1 ± 4.9 |
|  | Blood fed | 9 | 23.1 ± 6.7 | 3 | 25.0 ± 12.5 | 16 | 39.0 ± 7.6 | 2 | 14.3 ± 9.4 | 30 | 28.3 ± 4.4 |
|  | Half-gravid | 2 | 5.1 ± 3.5 | 2 | 16.7 ± 10.8 | 3 | 7.3 ± 4.1 | 3 | 21.4 ± 11.0 | 10 | 9.4 ± 2.8 |
|  | Gravid | 7 | 17.9 ± 6.1 | 1 | 8.3 ± 7.8 | 3 | 7.3 ± 4.1 | 4 | 28.6 ± 12.1 | 15 | 14.2 ± 3.4 |
|  | **Total** | **39** | **100.0 ± 0.0** | **12** | **100.0 ± 0.0** | **41** | **100.0 ± 0.0** | **14** | **100.0 ± 0.0** | **106** | **100.0 ± 0.0** |
| **Entente** | Unfed | 32 | 65.3 ± 6.8 | 6 | 46.2 ± 13.8 | 23 | 71.9 ± 7.9 | 6 | 54.5 ± 15.0 | 67 | 63.8 ± 4.7 |
|  | Blood fed | 5 | 10.2 ± 4.3 | 4 | 30.8 ± 12.8 | 4 | 12.5 ± 5.8 | 4 | 36.4 ± 14.5 | 17 | 16.2 ± 3.6 |
|  | Half-gravid | 7 | 14.3 ± 5.0 | 3 | 23.1 ± 11.7 | 2 | 6.3 ± 4.3 | 1 | 9.1 ± 8.8 | 13 | 12.4 ± 3.2 |
|  | Gravid | 5 | 10.2 ± 4.3 | 0 | 0.0 ± 0.0 | 3 | 9.4 ± 5.2 | 0 | 0.0 ± 0.0 | 8 | 7.6 ± 2.6 |
|  | **Total** | **49** | **100.0 ± 0.0** | **13** | **100.0 ± 0.0** | **32** | **100.0 ± 0.0** | **11** | **100.0 ± 0.0** | **105** | **100.0 ± 0.0** |
| **Gbagba** | Unfed | 16 | 47.1 ± 8.6 | 4 | 26.7 ± 11.4 | 25 | 59.5 ± 7.6 | 4 | 30.8 ± 12.8 | 49 | 47.1 ± 4.9 |
|  | Blood fed | 5 | 14.7 ± 6.1 | 5 | 33.3 ± 12.2 | 6 | 14.3 ± 5.2 | 4 | 30.8 ± 12.8 | 20 | 19.2 ± 3.9 |
|  | Half-gravid | 7 | 20.6 ± 6.9 | 4 | 26.7 ± 11.4 | 4 | 9.5 ± 4.5 | 3 | 23.1 ± 11.7 | 18 | 17.3 ± 3.7 |
|  | Gravid | 6 | 17.6 ± 6.5 | 2 | 13.3 ± 8.8 | 7 | 16.7 ± 5.8 | 2 | 15.4 ± 10.0 | 17 | 16.3 ± 3.6 |
|  | **Total** | **34** | **100.0 ± 0.0** | **15** | **100.0 ± 0.0** | **42** | **100.0 ± 0.0** | **13** | **100.0 ± 0.0** | **104** | **100.0 ± 0.0** |
| **Overall** | Unfed | 81 | 52.9 ± 4.0 | 21 | 42.0 ± 7.0 | 83 | 57.2 ± 4.1 | 18 | 37.5 ± 7.0 | 203 | 51.3 ± 2.5 |
|  | Blood fed | 27 | 17.6 ± 3.1 | 15 | 30.0 ± 6.5 | 33 | 22.8 ± 3.5 | 13 | 27.1 ± 6.4 | 88 | 22.2 ± 2.1 |
|  | Half-gravid | 20 | 13.1 ± 2.7 | 9 | 18.0 ± 5.4 | 13 | 9.0 ± 2.4 | 8 | 16.7 ± 5.4 | 50 | 12.6 ± 1.7 |
|  | Gravid | 25 | 16.3 ± 2.9 | 5 | 10.0 ± 4.2 | 16 | 11.0 ± 2.6 | 9 | 18.8 ± 5.6 | 55 | 13.9 ± 1.7 |
|  | **Total** | **153** | **100.0 ± 0.0** | **50** | **100.0 ± 0.0** | **145** | **100.0 ± 0.0** | **48** | **100.0 ± 0.0** | **396** | **100 ± 0.0** |
| %: percentage, n: number of *Aedes aegypti* mosquitoes, SE: standard error, SRS: short rainy season, LDS: long dry season, LRS: long rainy season, SDS: short dry season. | | | | | | | | | | | |
