# Supplementary material for: Assessing the ecological patterns of Aedes aegypti in areas with high arboviral risks in the large city of Abidjan, Côte d’Ivoire
Source: PLoS Negl Trop Dis. 2024 Nov 18;18(11):e0012647. doi: 10.1371/journal.pntd.0012647 (PMC11611265; doi:10.1371/journal.pntd.0012647)
Supplement: Attachment — Submitted filename: Comments.docx [file pntd.0012647.s013.docx]

68-69: Our findings revealed a significant presence of Aedes aegypti, indicating a high risk of Yellow Fever (YF) and Dengue (DEN) transmission across all study areas.

69-72: Aedes aegypti larvae were mostly breeding in tires, discarded containers and water storage containers. Meanwhile, elderly adult females were abundant, alongside a significant presence of both unfed host-seeking and blood-fed individuals in and around human residences.

72-76: We observed seasonal risk patterns, with permanent and high threats for dengue observed throughout both rainy and dry seasons over the entire year, alongside elevated risks of YF during both short and long rainy seasons across all study areas. Our findings provide insights into the ecology of *Aedes aegypti* and the epidemiology of DEN and YF, crucial for strategically targeting and controlling this vector in areas both affected and unaffected by disease outbreaks.

85: intensified instead of exacerbated.

88-89: In the West African region, over 27,000 arboviral cases were reported between 2007 and 2020, with the highest incidences and greatest burdens observed in major capital cities.

90-94: Given the absence of licensed vaccines for most arboviruses (excluding the yellow fever vaccine) and the lack of expanded routine prophylactic programs for *Ae.* vector control, surveillance of the primary vector *Ae. aegypti* is vital. It plays a crucial role in preventing, controlling, responding to, and preparing for arboviral outbreaks.

97-98: The ecological adaptability of *Ae. aegypti* allows this species to colonize various breeding sites in close proximity to human dwellings.

99-101: Since 1898 up until last year (2023), Côte d’Ivoire has experienced multiple outbreaks of YF and DEN, with a notably heightened resurgence and incidence in recent years, spanning from 2017 to 2023.

101-104: Despite the historical and present backgrounds, arboviruses remain uncontrolled, with ongoing resurgence of outbreaks posing a significant public health concern, particularly evident in the densely populated and highly urbanized city of Abidjan, Côte d’Ivoire.

108: *Ae*.

113-116: The interventions, largely unplanned due to the absence of robust data and accurate predictions, are urgently implemented in response to sudden arboviral outbreaks. However, they frequently yield limited and short-term impacts on local *Ae.* vector and arboviral control efforts.

General comments: After introducing 'Aedes' initially, it's advisable to consistently use the abbreviation 'Ae.' throughout the paper for brevity and consistency. Similarly, once 'dengue' has been abbreviated as 'DEN,' it should be consistently referred to as such for clarity and coherence in terminology usage. To enhance the quality of this paper, there is a need for improvement in the utilization of the English language.
