# Supplementary material for: Assessing the ecological patterns of Aedes aegypti in areas with high arboviral risks in the large city of Abidjan, Côte d’Ivoire
Source: PLoS Negl Trop Dis. 2024 Nov 18;18(11):e0012647. doi: 10.1371/journal.pntd.0012647 (PMC11611265; doi:10.1371/journal.pntd.0012647)
Supplement: Attachment — Submitted filename: Responses_Reviewer_R1.docx [file pntd.0012647.s014.docx]

**Ref: Submission ID PNTD-D-24-00163 - [EMID:312baae49fd6df57]**

**Adjobi *et al.* “Assessing the ecological patterns of *Aedes aegypti* in areas with high arboviral risks in the large city of Abidjan, Côte d’Ivoire”**

**Plos Neglected Tropical Diseases – Responses to Reviewers**

Abidjan, Basel, 11 August 2024

Dear Prof Olaf Horstick,

We were delighted to receive your e-mail dated 17 May 2024 in connection with the aforementioned manuscript. We thank you and the external reviewer very much indeed for providing a series of useful comments and suggestions, and we are grateful for your invitation to revise and re-submit our manuscript.

In the meantime, we have carefully reworked our piece in light of the editor and the reviewer reports. Below, please find our point-by-point response, clearly indicating how and where in the manuscript (line numbers) changes have been made. To further assist you in readily reviewing our changes made, we had the track-changes function on, We also upload a final clean version of our revised manuscript.

We feel that our revised manuscript has gained in clarity and quality and we very hope that it is now suitable for publication in *PLOS Neglected Tropical Diseases*.

Yours sincerely

Claver N’tayé Adjobi (on behalf of all authors)

**----------------------------------------------------------------------------------------------------------------**

**Editor comments**

*1) Your manuscript was reviewed by members of the editorial board and by several independent reviewers. In light of the reviews (below this email), we would like to invite the resubmission of a significantly-revised version that takes into account the reviewers' comments. We cannot make any decision about publication until we have seen the revised manuscript and your response to the reviewers' comments. Your revised manuscript is also likely to be sent to reviewers for further evaluation.*

**Authors’ response:** We thank the Editor and the external Reviewers very much indeed for scrutinizing our manuscript in such a timely manner and for inviting us to resubmit a revised version. In the meantime, we have carefully revised our piece and our responses are given below, using **blue ink**.

*2) 68-69: Our findings revealed a significant presence of Aedes aegypti, indicating a high risk of Yellow Fever (YF) and Dengue (DEN) transmission across all study areas.*

**Authors’ response:** We followed the editor’s suggestion, whilst effecting amendments according to this sentence above **(see revised manuscript, lines 83-85).**

*3) 69-72:* *Aedes aegypti larvae were mostly breeding in tires, discarded containers and water storage containers. Meanwhile, elderly adult females were abundant, alongside a significant presence of both unfed host-seeking and blood-fed individuals in and around human residences.*

**Authors’ response:** We revised this paragraph as per the Editor’s suggestion **(see revised manuscript, lines 85-88).**

*4) 72-76:* *We observed seasonal risk patterns, with permanent and high threats for dengue observed throughout both rainy and dry seasons over the entire year, alongside elevated risks of YF during both short and long rainy seasons across all study areas. Our findings provide insights into the ecology of Aedes aegypti and the epidemiology of DEN and YF, crucial for strategically targeting and controlling this vector in areas both affected and unaffected by disease outbreaks.*

**Authors’ response:** We revised this paragraph following the Editor’s suggestion (**see revised manuscript, lines 88-93).**

*5) 85:* *intensified instead of exacerbated.*

**Authors’ response:** As the Editor’s suggested, we replaced « exacerbated » with « intensified » **(see revised manuscript, line 109).**

*6) 88-89:* *In the West African region, over 27,000 arboviral cases were reported between 2007 and 2020, with the highest incidences and greatest burdens observed in major capital cities.*

**Authors’ response:** We followed the Editor suggestion and revised this sentence **(see revised manuscript, lines 111-113).**

*7) 90-94: Given the absence of licensed vaccines for most arboviruses (excluding the yellow fever vaccine) and the lack of expanded routine prophylactic programs for Ae. vector control, surveillance of the primary vector Ae. aegypti is vital. It plays a crucial role in preventing, controlling, responding to, and preparing for arboviral outbreaks.*

**Authors’ response:** We revised this section as the Editor suggested **(see revised manuscript, lines 116-120)**.

*8) 97-98:* *The ecological adaptability of Ae. aegypti allows this species to colonize various breeding sites in close proximity to human dwellings.*

**Authors’ response:** We revised this sentence according to the Editor suggestion **(see revised manuscript, lines 124-125).**

*9) 99-101:* *Since 1898 up until last year (2023), Côte d’Ivoire has experienced multiple outbreaks of YF and DEN, with a notably heightened resurgence and incidence in recent years, spanning from 2017 to 2023.*

**Authors’ response:** We revised this sentence as the Editor suggested. However, we replaced « last year (2023) » with « this year (2024) » and « from 2017 to 2023 » with from « 2017 up now (July 2024) » as Côte d’Ivoire is currently facing dengue outbreaks **(see revised manuscript, lines 126-128)**.

*10) 101-104:* *Despite the historical and present backgrounds, arboviruses remain uncontrolled, with ongoing resurgence of outbreaks posing a significant public health concern, particularly evident in the densely populated and highly urbanized city of Abidjan, Côte d’Ivoire.*

**Authors’ response:** We revised this sentence following the Editor suggestion **(see revised manuscript, lines 128-131)**.

*11) 108: Ae.*

**Authors’ response:** Please, we would like to keep « *Aedes* » instead of « *Ae.* », as *Aedes aegypti* is at the start of the sentence – Therefore no action was taken **(see revised manuscript, line 138).**

*12) 113-116: The interventions, largely unplanned due to the absence of robust data and accurate predictions, are urgently implemented in response to sudden arboviral outbreaks. However, they frequently yield limited and short-term impacts on local Ae. vector and arboviral control efforts.*

**Authors’ response:** We revised this section following the Editor suggestion **(see revised version manuscript lines 143-146)**. However, we replaced « *Ae*. » with « *Aedes* ». Indeed, as systematic role, one can abbreviate « *Aedes* » as « *Ae*. » only when writing the name of species, so « *Aedes aegypti* » becomes « *Ae. aegypti* ». The name of a genus alone cannot change and should remain « *Aedes* » **(see revised manuscript, lines 145)**.

*13) General comments: After introducing 'Aedes' initially, it's advisable to consistently use the abbreviation 'Ae.' throughout the paper for brevity and consistency.*

**Authors’ response:** We thank the Editor for this suggestion – Please note that this role is appropriate in general, but not in some specific cases such as the systematic or taxonomic name of genus and species. As indicated above, according to the taxonomic role, one can use the abbreviation « *Ae*. » to replace « *Aedes* » only when writing the name of a species, but the name of the genus « *Aedes* ». Therefore, we use « *Aedes* » when referring to *Aedes* genus and « *Ae. aegypti* » when referring to *Aedes aegypti* species through the paper. Moreover, if *Aedes aegypti* is at the start of a sentence, it should read « *Aedes aegypti* », but not « *Ae. aegypti* ».

*14) Similarly, once 'dengue' has been abbreviated as 'DEN,' it should be consistently referred to as such for clarity and coherence in terminology usage.*

**Authors’ response:** We absolutely agree with the Editor suggestion and replaced « dengue » with « DEN » through the paper **(see revised manuscript, lines 32, 65, 83, 89, 127, 136, 149, 150, 151, 152, 153, 154, 179, 182, 183, 185, 186, 188, 213, 214, 278, 292, 299, 448, 449, 451, 457, 458, 639, 642, 643, 691, 692, 697, 709, 718, 724, 725, 729, 731, 734, 736, 759, 779, 781,782, 784.**

**Reviewers’ comments**

**Comments: Reviewer#1**

*15) Reviewer #1: Results are completely presented but could be clearer (see general comments)*

**Authors’ response:** We thank the Reviewer #1 for going through our manuscript so carefully, and for providing a series of useful comments and suggestions. We followed the Reviewer #1’s comments and revised our manuscript accordingly.

**Reviewer#1:** Specific comments

*16) Lines 116, 118 are references available for the lack of success of control programmes*

**Authors’ response:** The lack of sustainable *Aedes* vector control can be seen via the multiple re-emergences of arboviral outbreaks in the same areas were *Aedes* vector control activities were implemented. We provided the references [11-17] **(see revised manuscript, line 128, line 146 and line 148)**.

*17) Line 128 little should read few*

**Authors’ response:** As the Reviewer #1 suggested, we replaced « little » with « few » **(see revised manuscript, line 158).**

*18) Line 134-5 it’s good to see a hypothesis being given but, given this hypothesis, what is the basis of the large differences in dengue rates among the different areas in the study between 2017-23?*

**Authors’ response:** We thank the Reviewer #1 for his question. Please, here we were assessing the entomological risks that are mainly based on the presence and abundance of *Aedes* vectors. We revised our hypothesis **(see revised manuscript, lines 165-167).**

*19) Line 179-181 What does ‘Aedes mosquito larvae and adults were sampled among 100 and 10 houses per study site and per survey, respectively.’ Mean i.e. were larvae sampled from 100 and adults from 10 houses? Rephrase to clarify.*

**Authors’ response:** We thank the Reviewer #1 for this suggestion. As the Reviewer #1 suggested, we rephrased the section to make it clearer and more comprehensible **(see revised manuscript, lines 215- 217).**

*20) Line 183-4* *investigated for larvae or larvae and adults?*

**Authors’ response:** *Aedes* larvae and adults were investigated. We provided this clarification **(see revised manuscript, line 221-222).**

*21) Line 216-8 was sampling only from the indoors of houses or also outdoors?*

**Authors’ response:** Sampling was done outdoors and indoors. We provided the clarification the on the sampling locations **(see revised manuscript, lines 251-253).**

*22) Line 333 was should read were*

**Authors’ response:** We thank the Reviewer#1 for drawing our attention in this typo. As the Reviewer#1 suggested, we replaced « was » with « were » **(see revised manuscript, line 354).**

*23) Line 339 appears to just repeat line 333 delete one instance to avoid confusion*

**Authors’ response:** The Reviewer#1 is right. We deleted the line 339**.**

*24) Line 341-44 I think this is saying that dry seasons positivity was higher than rainy seasons but can you clarify – text is not very clear and I may be misunderstanding*

**Authors’ response:** As the Reviewer #1 suggested, we reworded the sentences for more clarification **(see revised version manuscript, lines 367-371)**.

*25) Line 352 was should be were*

**Authors’ response:** As the Reviewer #1 suggested, we replaced « was » with « were » **(see revised manuscript, line 378).**

*26) Line 353-4 should read natural breeding sites*

**Authors’ response:** We followed the Reviewer #1’s suggestion, and replaced « naturals breeding site » with « natural breeding sites » **(see revised manuscript, line 380).**

*27) Line 385 Overall, Ae. aegypti positive breeding sites were observed inside of houses, but at a few proportion could read instead Overall, a small proportion of Ae. aegypti positive breeding sites were observed inside of houses*

**Authors’ response:** As the Reviewer #1 suggested, we replaced « Overall, *Ae. aegypti* positive breeding sites were observed inside of houses, but at a few proportion » with « Overall, a small proportion of *Ae. aegypti* positive breeding sites were observed inside of houses » **(see revised manuscript, lines 361-365).**

*28) Figure 2 and table 4 appear to contain almost exactly the same information therefore suggest to include only one (I prefer the table) as a main text illustration*

**Authors’ response:** We thank the Reviewer #1 for this suggestion. We partly agree with the Reviewer #1. However, we would like to maintain both Fig 2 and Table 4 as main illustrations. Indeed, Table contains data and details that are not in Fig 2. Fig contains all data that are in Table 2, but Fig 2 is more expressive and informative (visual). Similarly, Agha et al; included both Tabel and Figure reported the same information and data **(Agha et al., 2017; PLoS Negl Trop Dis, 11(8):e0005858).** Therefore, no actions have been taken - We just moved Fig 2 from Line 433 to Line 474 **(see revised manuscript, Lines 468, line 486, Table 4: line 466).**

*29) Line 490 was should be were*

**Authors’ response:** As per the Reviewer #1’s suggestion, we replaced « was » with « were » **(see revised manuscript, line 508).**

*30) Line 503-7 is this necessary when you have already said there is no significant difference?*

**Authors’ response:** The Reviewer #1 is right. There is no need to write this section - We therefore deleted this section**.**

*31) Lines 518-520 I don’t understand what is meant here because S1 Fig does not show seasonal variations*

**Authors’ response:** We agree with the Reviewer #1. This was a misleading – S1 Fig does not show seasonal variations. We thus deleted S1 Fig. **(see revised manuscript, Line 533).**

*32) Lines 556-565 just seem to repeat the information from Fig 4 and I think could be deleted Fig4 and 5a-d repeats information in Table 6 and whilst I think Fig 4 is Ok I don’t think non-significant variation warrants a four panel figure (Fig 5) – suggest to delete or move to supplementary*

**Authors’ response:** We agree with the Reviewer #1. As the Reviewer #1 suggested, we moved Fig 5 to supplementary as the supporting information S2 Fig **(see revised manuscript, lines 1038-1041 and S2 Fig)**. We kept Fig 4 as the main illustration **(see revised manuscript, lines 591)**. We moved Table 6 to supplementary as S4 Table **(see revised manuscript, lines 1073-1076 and S4 Table).**

**Discussion**

*33) Line 650 delete the word especially*

**Authors’ response:** As the reviewer #1 suggested, we deleted « especially » **(see revised manuscript, line 633)**.

*34) Line 652 et should be and*

**Authors’ response:** We are grateful to the Reviewer #1. We replaced « et » with « and » **(see revised manuscript, line 635)**.

*35) Line 656 delete brackets contents this is repeated a few lines later*

**Authors’ response:** As the reviewer #1 suggested, we deleted the brackets contents **(see revised manuscript, line 638)**.

*36) Line 663 replace the quasi-only with almost the only*

**Authors’ response:** We followed the reviewer #1’s suggestion, and replaced « the quasi-only » with « almost the only » **(see revised manuscript, line 645)**.

*37) Line 666 an should be a*

**Authors’ response:** As the Reviewer #1, we replaced « an » with « a » **(see revised manuscript, line 650)**.

*38) Line 683 Porto should read Puerto*

**Authors’ response:** As the Reviewer #1, we replaced « Porto » with « Puerto » **(see revised manuscript, line 665)**.

*39) Line 684 replace with ‘with water for various house cleaning tasks, to prevent water shortages’*

**Authors’ response:** As per the Reviewer #1’s suggestion, we replaced « with water for the various house cleanings, dishes in the house to prevent water shortages » with « with water for various house cleaning tasks, to prevent water shortages » **(see revised manuscript, lines 665-668)**.

*40) Line 690 plays should read play*

**Authors’ response:** Please, note that this sentence was deleted as the Reviewer #3 suggested.

*41) Line 690 second sentence could begin with ‘We observed that*

**Authors’ response:** Again, this sentence was deleted as the Reviewer #3 suggested.

*42) Line 698 suggest to delete … and therefore less …’ etc.*

**Authors’ response:** As the Reviewer suggested, we deleted the sentence.

*43) Line 705 replace, which with that*

**Authors’ response:** As the Reviewer #1 suggested, we deleted this sentence.

*44) Line 714 should read either tires or a tire*

**Authors’ response:** We followed the reviewer #1’s suggestion, we replaced « tire » with « tires » **(see revised manuscript, line 681)**.

*45) Line 727 add the before rainy and suggest to delete second sentence*

**Authors’ response:** We followed the reviewer #1’s suggestion, so we have deleted the sentence.

*46) Lines 728-31 need checking and rewriting*

**Authors’ response:** As the Reviewer #1 suggested, we have deleted these sentences.

*47) Line 741-744 this is a key result for control and needs emphasising e.g. in abstract*

**Authors’ response:** We thank the reviewer#1 for this suggestion. As the Reviewer suggested, we added this sentence into the Abstract section as a part of the conclusion **(see revised manuscript, lines 64-66 and lines 779-780)**.

*48) Line 752-3 it would be good to give more details about what these awareness campaigns constitute*

**Authors’ response:** We are grateful to the Reviewer#1 for these useful suggestions. We gave more details about the awareness campaigns. **(see revised manuscript, lines 699-707)**.

*49) Line 766 move ‘so-called’ to before epidemic-free (or delete)*

**Authors’ response:** As the Reviewer #1 suggested, we removed « so-called » before epidemic-free and we have deleted the sentences.

*50) Line 771 high relative to what?*

**Authors’ response:** Please, note that we are not comparing the AHHs to an established index. As AHHs were 5.72 *Aedes*/house/hour (e.g. with 12.3 *Aedes*/house/hour in Entente during SRS) **(see revised manuscript, Table 5)**, we consider that AHHs are high based on our experience**.**

*51) Line 777 isn’t the sampling only conducted within houses?*

**Authors’ response:** Please, note that *Aedes* sampling was conducted in domestic (indoors and outdoors of houses) premises and in peri-domestic premises as described in the Methods section **(see revised manuscript, lines 219-220)**.

*52) Line 779-780 you did not type the bloodmeals so you are assuming (probably correctly) a high HBI but this assumption needs justifying with respect to literature*

**Authors’ response:** We thank the Reviewer#1 for this suggestion. We provide the reference of a study that typed the blood-meals in a West African city, Ouagadougou, Burkina Faso that is similar to Abidjan city, and the authors of this study reported that the human blood index (HBMI) was >90% (Badolo et al. PLoS Negl Trop 2022 Jul 6;16(7):e0010059) **(see revised manuscript, lines 718-721)**. We add HBI assessment as a limitation of this study and suggested this for further analyses **(see revised manuscript, lines 735-737)**.

*53) Line 785 suggests rather than revealed*

**Authors’ response:** This sentence was deleted.

*54) Line 814 delete ‘with differential of arboviral indices*

**Authors’ response:** We followed the Reviewer #1’s suggestion, and deleted « with differential of arboviral indices »

*55) Lines 817-820 need checking and rewriting*

**Authors’ response:** As the Reviewer #1 suggested, we checked and reworded the conclusion section **(see revised manuscript, lines 773-787)**.

*56) In this study the ecology of Aedes (primarily aegypti) was assessed in four cites within Abidjan encompassing high and low transmission area across four seasons over one year in 2019-2020. This is a very large dataset and the key result is probably the lack of entomological difference among areas of the city which differ markedly in arbovirus case rates, and limited seasonal variation. This is an important finding for control moving away from reactive entomological control operations to more proactive preventative control when possible. It would be good however for the authors to comment more on any socio-epidemiological factors which might explain the differences in case rates. If entomological indices are not predictive of current patterns are there other factors at play which might be important. This element is absent at present. The Introduction and Methods are well written but the Results are quite densely written with apparently unnecessary repetition of statistics among tables figures and text which makes them quite hard to follow in places. I have made some suggestions below to try to help streamline but I think it would be helpful if the authors checked through and tried to remove unnecessary repetition. Also in several places results are referred to as being higher or lower then followed by a test results which shows no difference; this is better avoided. The Discussion is rather lengthy but does cover all key points however the conclusion needs rewriting.*

**Authors’ response:** We thank very much the Reviewer #1 for the positive overall appraisals of our work. As suggested by the Reviewer #1, we provide some socio-epidemiological factors that might explain the difference in dengue and yellow fever case rates **(see revised manuscript, lines 742-752)**. Additionally, we deleted unnecessary repetition of statistics. We also moved some figures to supporting information – For instance, we changed Fig 5 to the supporting information S3 Fig **(see revised manuscript, lines 1043-1045 and S3 Fig)**. We moved Table 6 to the supporting information S1 Table **(see revised manuscript, lines 1047-1056 and S1 Table).** We added the section showing the limitations of the study **(see revised manuscript, lines 733-742)**. We highlighted the study relevance for public health **(see revised manuscript, lines 754-770)**. We revised the Conclusion **(see revised manuscript, lines 773-787)**.

**Reviewer#2**

*57) The paper's methodology looks good overall. The study's goals are clear, and there's a testable hypothesis. The design of the study seems right for what they're trying to find out, and they've described the group they're studying well. The statistics they've used make sense. There don't seem to be any ethical or regulatory issues either. So, the methodology seems solid.*

**Authors’ response:** We thank the Reviewer #2 for the positive overall appraisals of our work.

*58) In the results section, it seems like they followed the analysis plan. But they could improve the English language for better understanding. The results are clear and complete, though they should work on making the figures (like tables and images) clearer. So, they need to brush up on their English and improve the quality of their figures*

**Authors’ response:** We thank the Reviewer #2 for his/her positive comments. We revised manuscript to the English – Additionally, we followed the Reviewer #1’s suggestions that substantially help us to revise and improve the English in many places. We also improved the quality of figures and checked the quality using PACE.

*59) The paper's conclusions seem based on the data, but the English in that part could be better. They did not mention the limitations. They need to better address why this research matters for public health. So, they need to improve the conclusion, mention limitations, and discuss public health relevance*

**Authors’ response:** We followed the Reviewer #2’s suggestion, and we rewrote to improve the English in several places. As the Reviewer #2 suggested, we improved the conclusion **(see revised manuscript, lines 773-787)**, mentioned limitations **(see revised manuscript, lines 733-742)**, and discussed public health relevance **(see revised manuscript, lines 754-770)**.

**Reviewer#3**

*60) Line 31. I’m not sure that it’s outbreak of Aedes mosquito-borne arbovirus. Dengue and yellow fever are diseases or viruses*

**Authors’ response:** We thank the reviewer #3 for his/her helpful comments and suggestions. We followed the Reviewer #3’s comments and revised this section replacing « arboviruses » with « arboviral diseases » **(see revised manuscript, lines 32)**.

**Background**

*61) Line 82. Aedes-borne viruses?*

**Authors’ response:** As indicated above, we replaced « arboviruses » with « arboviral diseases » **(see revised manuscript, line 105)**.

*62) Lines 88-89. It would be good to update this information. In 2023 146 878 cases of dengue were reported in Burkina-Faso with 688 deaths.*

**Authors’ response:** As the Reviewer #3 suggested, we updated the information related to the epidemiology of dengue in West Africa in 2023 **(see revised manuscript, lines 113-116)**.

*63) Line 93. arboviral outbreaks????*

**Authors’ response:** Please, note that we revised this sentence as suggested by the Reviewer #1 **(see revised manuscript, line 120)**.

*64) Lines 93-94. “Aedes aegypti is a key vector of arboviruses in Africa”. Reference is needed.*

**Authors’ response :** As the Reviewer #3 suggested, we added a reference [1] **(see revised manuscript, lines 120).**

*65) Line 99-101. Reference*

**Authors’ response :** As the Reviewer #3 suggested, we added references [11-17] **(see revised manuscript, line 128).**

*66) One sentence should be added in the background about dengue control.*

**Authors’ response:** Please, note that dengue control in Côte d’Ivoire is mainly based on *Aedes* vector control, and we had already added this information **(see revised manuscript, lines 139-146)**.

*67) Lines 108-109. “Aedes aegypti in Abidjan are resistant to most 108 insecticides used for their control [13]”. Have you assessed the insecticide resistance to Aedes in this manuscript?*

**Authors’ response:** Please note that we did not assess the insecticide resistance in *Aedes* in this manuscript. This is related to the second sentence about the use of insecticides for *Aedes* vector control that often resulted in limited and short-term effects **(see revised manuscript, lines 139-142)**.

*68) Line 148. Reference is needed*

**Authors’ response :** As the Reviewer #3 suggested, we added a reference [11-17] **(see revised manuscript, lines 180).**

*69) The level of urbanization is similar in the four municipalities selected? Type of habitation? Vegetation? Water supplies? Waste management?*

**Authors’ response:** We thank the Reviewer #3 for these important questions for better understanding of the ecology of the local *Ae. aegypti* in the four study sites. As per observation, we can confirm that the four study sites had roughly and apparently similar ecological and geographical characteristics. Unfortunately, we did not carry out specific surveys to assess the level of urbanization, the type of habitation, the vegetation, the water supply and the waste management in the four study sites. Therefore, we added these key aspects as a limitation of our current study in the Discussion section **(see revised manuscript, lines 747-751**.

*70) How were households selected?*

**Authors’ response:** Please note that the households were randomly from the total of houses that were labeled with unique numbers **(see revised manuscript, lines 219-222)**.

*71) There is a difference between households and concession or house?*

**Authors’ response:** Please note that there is a difference between house, household and concession. The house referred to a building. The household refers to the group of people living in one or several houses under a head. The concession is the groups of households with several households in the same courtyard. In our study the encountered the three types of habitation, but it was sometimes difficult to find the limits or boundaries of households among certain concessions. However, we focused on households and the main building was selected in each selected households for sampling **(see revised manuscript, lines 216-220)**.

*72) Line 192. “Aedes mosquito larvae (larvae and pupae)”?????*

**Authors’ response:** We replaced « larvae » with « immatures » section **(see revised manuscript, line 227)**.

*73) Culex tigripes???? Please change this by Lutzia tigripes.*

**Authors’ response:** As the Reviewer #3 suggested, we replaced « *Culex tigripes* » with « *Lutzia tigripes* », throughout all the manuscript **(see revised manuscript, lines 230, lines 232-233, line 330 and Table 1)**

*74) Other Aedes species were found? if yes, please explain the process to establish the proportion of each species.*

**Authors’ response:** Please, note that we collected two specimens of *Aedes palpalis* (0,01%, 2/25,658) in one study site (Gbagba) **(see revised manuscript, lines 339, Table 1)**,

*75) Line 216. Domestic and peridomestic premise ???? it’is indoor/outdoor?? It is not clear enough*

**Authors’ response:** Please note that we sampled *Aedes* indoors and outdoors of 10 selected houses in domestic premises and surrounding peridomestic premises **(see revised manuscript, lines 219-220)**.

*76) Line 217. Why was this period selected for adults collection? What was target? Resting mosquitoes?*

**Authors’ response:** Please, note that we selected these periods (06:00 a.m. to 09:00 a.m. and in the afternoon from 3:00 p.m. to 6:00 p.m.) for adult collections because *Aedes aegypti* is diurnal mosquito – We were collecting the resting individuals **(see revised manuscript, lines 253-255)**.

*77) Line 222. Why 20 individuals?*

**Authors’ response:** Please, note that we put up to 20 individuals per cup to avoid overcrowding and minimize mortality during the rearing **(see revised manuscript, lines 259-260)**.

*78) Line 303. Not clear. Across the manuscript there is sometime confusion between disease and pathogen*

**Authors’ response:** As the Reviewer #3 suggested, we replaced « malaria » with « *Plasmodium* malaria parasites » **(see revised manuscript, line 342)**.

*79) Table 1. it’s possible to determine female and male from larvae*

**Authors’ response:** Please, note that larvae were identified at adult stage after rearing **(see revised manuscript, lines 259-264)**. Therefore, we could determine female and male of larvae from the emerged adults issued from larvae as mosquito larvae cannot change sex during or after rearing.

*80) Tigripes belong to the genus Lutzia. Please correct it across the manuscript.*

**Authors’ response:** We thank the Reviewer #3 for this update. As we indicated above, we replaced « *Culex tigripes* » with « *Lutzia tigripes* » throughout in all the revised manuscript **(see revised manuscript, lines 230, lines 232-233, line 330 and Table 1).**

*81) What is the potential larval breeding container?*

**Authors’ response:** Please, note that we defined « potential breeding containers » as wet breeding sites likely to contain *Aedes* immatures (larvae and/or adults)

**Results**

*82) What was the mainly found and most productive larval habitat in rainy season? Or dry season?*

**Authors’ response:** Please note that the most productive larval habitats were water storage containers in short rainy season (43.7%, n = 12,611), tires in long dry season (LDS) (44.7%, n = 5,886) and long dry season (LDS) (33.7%, 12,213) and water storage containers in short dry season (SDS) (46.1%, n = 2,402) **(see revised manuscript, Lines 414-416, Table 3).**

*83) What was the proportion of Ae. aegypti collected indoor/outdoor?*

**Authors’ response:** As the Reviewer #3, we provided the proportions of *Ae. aegypti* collected outdoors (93.4%) and indoors (6.6%) of houses (**see revised manuscript, lines (541-547)**.

*84) Why the origin of blood meals ingested by Ae. aegypti was not established? I suggested to the authors to add this important information in the manuscript.*

**Authors’ response:** We thank very much the Reviewer #3 for this important suggestion. We are afraid - We did not determine the origin of the bloodmeals of *Ae. aegypti* in our present study due to the lack of funding. As the Reviewer #3, we will analyze molecularly *Aedes* bloodmeals to determine their sources and hosts in our future study. In the meantime, we added the assessment of the origin of *Aedes* bloodmeals a limitation of our study (**see revised manuscript, lines 735-737)**.

**Discussion**

*85) Lines 646-648. Reference*

**Authors’ response:** As the Reviewer #3 suggested, we added the references [11-15, 17, 30-33] **(see revised manuscript, line 630)**.

*86) Line 660 replace community by population*

**Authors’ response:** As the Reviewer #3 suggested, we replaced « communities » with « populations » **(see revised manuscript, line 642)**.

*87) Lines 664-666. This is true when Ae. albopictus is absent in the location. Please this sentence needs to be reformulated.*

**Authors’ response:** We agree with the Reviewer #3. We thus included this precision « … where *Aedes* *albopictus* are absent or rare … » **(see revised manuscript, lines 646-650)**.

*88) What does it mean strong desiccation?*

**Authors’ response:** We reworded the sentence. We wanted to write that *Aedes* eggs are resistant to desiccation and can survive form drought and then hatch during the rainy seasons **(see revised manuscript, lines 671-673)**.

*89) Please replace entomological risk by potential entomological risk*

**Authors’ response:** As suggested by the Reviewer, we replaced « entomological risk» by « potential entomological risk» **(see revised manuscript, lines 690).**

*90) Lines 676-680. What is the duration between rainy and dry season?*

**Authors’ response:** Please, note that the duration between rainy and dry seasons in Abidjan (Côte d’Ivoire) vary: the short dry seasons (2 month: August-September) and the long dry seasons (4 months: December-March) as described in the Methods section **(see revised manuscript, lines 189-192)**.

*91) Lines 683-687. speculation*

**Authors’ response:** We thank the Reviewer #3 for this comment. Our current study did not report the storage of water, but the high infestation of water storage containers with *Aedes* larvae. Additionally, previous and some studies being conducted in the same or similar study areas in Abidjan reported that local populations store water for long duration and Aedes larvae were found in the uncovered and unmanaged water storage containers as reported in Zahouli et al. 2017 PLoS Negl Trop dis. We reworded the sentences **(see revised manuscript, lines 666-669)**,

*92) Please could you discuss the results of the typology of larval habitats considering the observation found elsewhere in Africa and out of Africa?*

**Authors’ response:** As the Reviewer #3 suggested, we discussed the result on the typology of breeding sites as agreed by the Reviewer **(see revised manuscript, lines 655-665).**

*93) I would like to draw the authors' attention to the fact that the presence of the vector alone is not enough for there to be an epidemic. It should also be noted that the vectors incriminated here have diurnal activity and people can receive bites outside their place of residence. Please consider this in the discussion*

**Authors’ response:** As the Reviewer #3 suggested, we added a section on the lack correlation between the presence of *Aedes* and the occurrence of arboviral epidemics **(see revised manuscript, lines 729-733).**

*94) Line 692-693. “The high positivity of tires is due to the high presence of tires and their use by local communities”. They are using tires to do what?*

**Authors’ response:** We followed the Reviewer #3’s suggestion, and provided details on the use of the tires. Indeed, the car tires were used for the production of the local dish “Attiéké”, roofing, decorating and selling as reported in Zahouli et al. [18] **(see revised manuscript, lines 673-676).**

*95) Lines 803-804. reference*

**Authors’ response:** We deleted this section in the Discussion section as it has been included with the references [11-17] into the introduction section **(see revised manuscript, line 148)**.

*96) One sentence or paragraph on the absence of Ae. albopictus in Abidjan will be helpful especially since his presence was notified in this city in 2010 (Konan et al. 2013).*

**Authors’ response:** As the Reviewer #3 suggested, we added two sentences on the absence of *Ae. albopictus* in our study areas. *Aedes albopictus* was not sampled in our current study, but was recorded in Abidjan in 2010 [34] and 2014 [18] **(see revised manuscript, lines 645-650)**.

**Additional inputs from the Authors**

**Authors’ response:** We added one section on Ae. aegypti resting behaviors **(see revised manuscript, lines 541-547)**.

**Authors’ response:** We added three additional Supporting Tables (S 1 Table, S2 Table and S3 Table) on the abundance and behaviors of adult populations of *Aedes aegypti* **(see revised manuscript, lines 1047-1056, lines 1058-1067, lines 1069-1071, S1 Table, S2 Table and S3 table)**.
