# Supplementary material for: Assessing the ecological patterns of Aedes aegypti in areas with high arboviral risks in the large city of Abidjan, Côte d’Ivoire
Source: PLoS Negl Trop Dis. 2024 Nov 18;18(11):e0012647. doi: 10.1371/journal.pntd.0012647 (PMC11611265; doi:10.1371/journal.pntd.0012647)
Supplement: Attachment — Submitted filename: MB- review.docx [file pntd.0012647.s015.docx]

-Avoid abbreviations in the abstract.

- After the first use the word *Aedes* you should use the abbreviation *Ae*. (check the whole text).

- More information is needed on the identification process of the sampled mosquitoes.

- Please give me the exact reference that you used for the thresholds of WHO that define the risk of disease in relation to the no. of larvae/adults that you collected.

- The discussion part should include weaknesses of the current study and future ideas.

- Throughout the manuscript more references should be added to justify all the information that you included.

- English need to be improved.

Some of the corrections that are needed

Line 85 *Ae. aegypti* in italics

Line 105-108 *Aedes* mosquito-borne arboviral diseases such as dengue (DEN), yellow fever (YF), chikungunya (CHIK) and Zika (ZIK), pose a significant threat to over 831 million people, representing 70% of the population in sub- Saharan Africa [1]. These diseases have the heaviest public health and socio-economic impacts in highly urbanized cities [2,3].

Line 115 stands out, instead of emerges

Line 116-119 re-phrase: In the absence of licensed vaccines for most arboviruses (except for the YF vaccine) and the lack of widespread routine prophylactic programs for controlling *Ae.* vectors, surveillance of the primary vector, *Ae. aegypti*, is crucial.

Line 119-120 this phrase comes in contrast with the previous sentence. No programs for controlling aedes vectors but surveillance for Aedes aegypti crucial for preventing, controlling, responding to, and preparing for to prevent arboviral outbreaks?

Line 121-124 *Ae. aegypti*, a key vector of arboviruses in Africa, can transmit over 50 viruses to humans and exhibits highly anthropophagic behavior, dwelling in and around human habitats—such as domestic and peridomestic premises—where females predominantly feed on humans and breed in man-made container.

Line 126-129 Since 1898 up to the present year (2024), Côte d’Ivoire has experienced multiple outbreaks of YF and DEN, with a notable resurgence and increased incidence in recent years, particularly from 2017 to the present.

Line 127: delete heightened and add increase before incidence. Add particularly and delete spanning

Line 128: To present (delete up to now)

Line 132- 134: (Rephrase) The city is marked by rapid, uncontrolled urbanization and complex land cover changes, driven by poor urban planning and limited environmental and sanitation management services.

Line 137-138 (Rephrase) This is largely due to restricted financial investments, along with limited operational resources and technical capacities.

Line 139-143 (Rephrase) The government’s response to outbreaks, led by the National Institute of Public Hygiene (NIPH) under the Ministry of Health (MoH) of Côte d’Ivoire, primarily relies on sporadic insecticide space spraying targeting adult *Ae.* mosquitoes, along with systematic removal, physical destruction, and/or treatment of larval breeding sites.

Line 152 delete the word “there”

Line 152-153 better to combine the two sentences in one.

Line 159 *Ae.*

Line 180-186 (Rephrase) In this study, four urban municipalities were selected based on their epidemiological backgrounds and incidences of dengue (DEN) and yellow fever (YF): Bingerville (5° 21’ N; 3° 54’ W) and Cocody (5° 20’ N; 3° 58’ W), located in the Cocody-Bingerville health district where DEN and YF cases are regularly reported; Treichville (5° 19’ N; 4° 01’ W), situated in the Marcory-Treichville district with few recorded cases of DEN and YF; and Yopougon (5° 20’ N; 4° 00’ W), where DEN and YF cases are rare.

Line 188: reported between 2017 to 2024, according to hospital data.

Lines 210- 215 I think this sentence repeats the lines 180-188. Delete or rephrase.

Linse 222-223 don’t understand the meaning of this sentence.

Lines 329-330: check the percentages of each genus

Lines 329-330: I would suggest to present the number of each genus differently, it is not necessary to include the total number of mosquitoes each time, but you could present the number of males and females you collected for each genus and delete the ( before the word *Aedes*.

Line 334 you have already share this information (line 329) about the proportion of *Aedes* genus

Line 337 you have already share this information (lines 330-331)

Lines 339-343 Other medically important non-*Aedes* species, such as *Culex quinquefasciatus* (2.65%, 2,298/86,796), a vector of arboviruses, and *Anopheles gambiae* s.l. (0.16%, 136/86,796), a vector of *Plasmodium spp,* were also collected across the four study areas, though in relatively low proportions.

Lines 343-346 this information (the developmental stage of the collected mosquito) should be given from the beginning of the results section.

Table 1: maybe delete the line with the subtotal, it is confusing.
