# Supplementary material for: Assessing the ecological patterns of Aedes aegypti in areas with high arboviral risks in the large city of Abidjan, Côte d’Ivoire
Source: PLoS Negl Trop Dis. 2024 Nov 18;18(11):e0012647. doi: 10.1371/journal.pntd.0012647 (PMC11611265; doi:10.1371/journal.pntd.0012647)
Supplement: Attachment — Submitted filename: Responses_Reviewer_R2.docx [file pntd.0012647.s016.docx]

**Ref: Submission ID PNTD-D-24-00163 - [EMID:312baae49fd6df57]**

**Adjobi *et al.* “Assessing the ecological patterns of *Aedes aegypti* in areas with high arboviral risks in the large city of Abidjan, Côte d’Ivoire”**

**Plos Neglected Tropical Diseases – Responses to Reviewers**

Abidjan, Basel, 05 October 2024

Dear Prof Olaf Horstick,

We were delighted to receive your e-mail dated 12 September 2024 in connection with the aforementioned manuscript. We thank you and the external reviewer very much indeed for providing a series of useful comments and suggestions, and we are grateful for your invitation to revise and re-submit our manuscript.

In the meantime, we have carefully reworked our piece in light of the editor and the reviewer reports. Below, please find our point-by-point response, clearly indicating how and where in the manuscript (line numbers) changes have been made. To further assist you in readily reviewing our changes made, we had the track-changes function on, We also upload a final clean version of our revised manuscript.

We feel that our revised manuscript has gained in clarity and quality and we very hope that it is now suitable for publication in *PLOS Neglected Tropical Diseases*.

Yours sincerely

Claver N’tayé Adjobi (on behalf of all authors)

**----------------------------------------------------------------------------------------------------------------**

**Reviewers’ comments**

**Comments: Reviewer#1**

**Summary and General** **Comments**

*1) Reviewer #1: The authors have done a good job with the revision and I just have a few minor comments (below)*

**Authors’ response:** We would like to thank the Reviewer #1 for his encouragement and we will endeavor to consider any suggestions.

*2)* **Comment on:** *Authors’ response: We thank the Reviewer #1 for his question. Please, here we were assessing the entomological risks that are mainly based on the presence and abundance of Aedes vectors. We revised our hypothesis (see revised manuscript, lines 165-167). Suggest to include the word entomological before risks to clarify the hypothesis and avoid this sounding like a tautology*

**Authors’ response:** As suggested by the Reviewer #1, we added « entomological » before risks (see revised manuscript, line 166).

*3) Comment on: 50) Line 771 high relative to what? Authors’ response: Please, note that we are not comparing the AHHs to an established index.* *As AHHs were 5.72 Aedes/house/hour (e.g. with 12.3 Aedes/house/hour in Entente during SRS) (see revised manuscript, Table 5), we consider that AHHs are high based on our experience. I think the authors need to add this statement or provide some additional support here to make it clear that this is to some extent at least a subjective assessment*

**Authors’ response:** As the Reviewer #1 for greater clarity, we deleted «were high and » (see revised manuscript, lines 733-734).

***Line numbers refer to manuscript with track changes***

4) *Line 164 delete 19 July*

**Authors’ response:** We thank the Reviewer #1 for this suggestion. As the Reviewer #1 suggested, we deleted « 19 July » (see revised manuscript, line 128).

5) *Line 502-503 missing ‘of’*

**Authors’ response:** We thank the Reviewer #1 for his comment. We have added « of » to the sentence (see revised manuscript, line 420).

6) *Line 779– delete ‘these’*

**Authors’ response:** We thank the Reviewer #1 for his comment. We deleted « these » in the sentences (see revised manuscript, line 643).

*7) Line 780-782 Given the uninformativeness of the Stegomyia indices and adult abundances (in Discussion and Conclusion), how is the assessment of medium to high levels of risk of transmission assessed? Same comment about lines 905-908 and 913-4. Note that the word potentially is added to line 909 and it would seem appropriate to add this or a similar qualifier elsewhere*

**Authors’ response:** The assessment of the medium to high risk level for dengue and yellow fever transmission was obtained using the larval density scale established by the WHO, as shown in the Data Analysis section of the manuscript from lines 294 to 307. Furthermore, these same risk levels were applied by Padonou *et al*., 2020 **(https://doi.org/10.1186/s41182-020-00207-w),** and Agha *et al*., 2017 **(https://doi.org/ 10.1371/journal.pntd.0005858)**

*8)* *Line 842-83. High positivity indicates a high proportion of tires infested rather than a large number of total breeding sites that were tires – may want to rephrase this for clarity*

**Authors’ response:** As suggested by the Reviewer #1, we have reworded the sentence “The high *Aedes*-positivity of tires indicates a high proportion of tires infested with *Ae. aegypti* immatures” (see revised manuscript, lines 693 - 694).

*9) Line 962 the word probably doesn’t fit here*

**Authors’ response:** As the Reviewer #1 suggested, we deleted « probably imply the » and replaced it by “be due to their“ (see revised manuscript, line 746).

*10) Line 965 delete the word any*

**Authors’ response:** We followed the Reviewer #1’s suggestion and deleted « any » (see revised manuscript, lines 747).

*11) Line 970 should ‘T’ be This?*

**Authors’ response:** As the Reviewer #1 suggested, we corrected the sentences (see revised manuscript, lines 750 - 751).

*12) Line 1019 are should be is*

**Authors’ response:** We thank the Reviewer #1 for his comments. We replaced « are » by « is » (see revised manuscript, lines 772).

**Reviewer#2**

*13) The results are presented, but additional clarity is needed, particularly in relation to the identification of the sampled mosquitoes. More information on the specific identification techniques and criteria used should be included to support the conclusions. Furthermore, providing the exact WHO references for the risk thresholds would enhance the validity of the results and their interpretation. Tables need to be more clear. Improving the organization and detail of the tables would strengthen the presentation of the data.*

**Authors’ response:** We thank the Reviewer #2 for these very helpful comments and suggestions. We followed revised the specific sections accordingly. We hope that our revised manuscript has gained in more clarity.

*14) Avoid abbreviations in the abstract.*

**Authors’ response:** We thank the Reviewer #2 for this suggestion. Please, note that given the word number limitation in the Abstract section, we used the abbreviations to respect this instruction length. Moreover, the Abstract of articles published recently in PloS Neglected Tropical Diseases contains abbreviations – as seen in **Srisawat et al. 2024 (PLoS Negl Trop Dis 2024 Mar; 18(3): e0012060 ; doi:** [**10.1371/journal.pntd.0012060**](https://doi.org/10.1371%2Fjournal.pntd.0012060)**)**. Therefore, we have made no change regarding the abbreviations in the Abstract.

*15) After the first use the word Aedes you should use the abbreviation Ae. (check the whole text).*

**Authors’ response:** As the Reviewer #2, we replaced “*Aedes*” with “*Ae.*” and this was done in the whole text. However, this change was applied only in the name of species (*Aedes aegypti* become *Ae. aegypti*), but not in the name of the genus (*Aedes* remains *Aedes*).

*16) More information is needed on the identification process of the sampled mosquitoes.*

**Authors’ response:** We have noted the species identification keys used (see line 267) and added the criteria we used. The species identification was based on the morphological aspect, including the color of the scutum, the appearance of the legs and wings, the shape of the thorax and the proboscis of the specimens (See revised manuscript, lines 266 -267).

*17) Please give me the exact reference that you used for the thresholds of WHO that define the risk of disease in relation to the no. of larvae/adults that you collected.*

**Authors’ response:** We provided the exact references for the *Aedes aegypti* larval density indices used to assess the *Stegomyia* indices for potential dengue and yellow fever virus transmission risks (see bibliographic references 30 and 31). Furthermore, these same risk indices and risk levels were applied by Padonou *et al*., 2020 **(https://doi.org/10.1186/s41182-020-00207-w),** and Agha *et al*., 2017 **(https://doi.org/ 10.1371/journal.pntd.0005858).**

*18) The discussion part should include weaknesses of the current study and future ideas.*

**Authors’ response:** We followed the Reviewer #2’s suggestion. We mentioned weaknesses in the discussion **(see revised manuscript, lines 757-768) and for the future ideas (see revised manuscript 789 -796)**

*19) Throughout the manuscript more references should be added to justify all the information that you included*

**Authors’ response:** We thank the Reviewer #2. Please, note that manuscript has 65 references that is enough. Recent articles published in PLoS Negl Trop Dis have less than 65 references (Dutra et al., [PLoS Negl Trop Dis.](https://www.ncbi.nlm.nih.gov/pmc/articles/PMC11414913/) 2024 Sep; 18(9): e0012482 (56 references); Cruz et al., [PLoS Negl Trop Dis.](https://www.ncbi.nlm.nih.gov/pmc/articles/PMC11296630/) 2024 Jul; 18(7): e0011603 (60 refrences); Wang et al., 2024, [PLoS Negl Trop Dis.](https://www.ncbi.nlm.nih.gov/pmc/articles/PMC11081495/) 2024 Apr; 18(4): e0012158).

*20) English need to be improved.*

**Authors’ response:** As the Reviewer #2’s suggested, we have improved English.

**Some of the corrections that are needed**

*21) Line 85 Ae. aegypti in italics*

**Authors’ response:** We thank the Reviewer #2 for his comments. We have written « *Ae. aegypti* » in italics (see revised manuscript, line 85).

*22)* *Line 105-108 Aedes mosquito-borne arboviral diseases such as dengue (DEN), yellow fever (YF), chikungunya (CHIK) and Zika (ZIK), pose a significant threat to over 831 million people, representing 70% of the population in sub- Saharan Africa [1]. These diseases have the heaviest public health and socio-economic impacts in highly urbanized cities [2,3].*

**Authors’ response:** We have considered the Reviewer #2’s corrections (see revised manuscript, lines 105 -108).

*23) Line 115 stands out, instead of emerges*

**Authors’ response:** As the Reviewer #2 suggested, we replaced emerges by stands out (see revised manuscript, line 116).

*24) Line 116-119 re-phrase: In the absence of licensed vaccines for most arboviruses (except for the YF vaccine) and the lack of widespread routine prophylactic programs for controlling Ae. vectors, surveillance of the primary vector, Ae. aegypti, is crucial.*

**Authors’ response:** We have considered the Reviewer #2’s suggestion (see revised manuscript, lines 117 – 120)

*25) Line 119-120 this phrase comes in contrast with the previous sentence. No programs for controlling Aedes vectors but surveillance for Aedes aegypti crucial for preventing, controlling, responding to, and preparing for to prevent arboviral outbreaks?*

**Authors’ response:** We rewrote the sentences according the suggestions of the Reviewer #2 (see revised manuscript, lines 119 -121).

*26) Line 121-124 Ae. aegypti, a key vector of arboviruses in Africa, can transmit over 50 viruses to humans and exhibits highly anthropophagic behavior, dwelling in and around human habitats—such as domestic and peridomestic premises—where females predominantly feed on humans and breed in man-made container.*

**Authors’ response:** We have considered the Reviewer #2’s suggestions (see revised manuscript, lines 121 – 124)

*27) Line 126-129 Since 1898 up to the present year (2024), Côte d’Ivoire has experienced multiple outbreaks of YF and DEN, with a notable resurgence and increased incidence in recent years, particularly from 2017 to the present.*

**Authors’ response:** As the reviewer suggested, we have considered the Reviewer #2’s suggestions (see revised manuscript, lines127 – 129)

*28) Line 127: delete heightened and add increase before incidence. Add particularly and delete spanning*

**Authors’ response:** We have done this in response 24) (see revised manuscript, line 128 - 129).

*29) Line 128: To present (delete up to now)*

**Authors’ response:** As the Reviewer #2 suggested, we have done this (see revised manuscript, line 129).

*30) Line 132- 134: (Rephrase) The city is marked by rapid, uncontrolled urbanization and complex land cover changes, driven by poor urban planning and limited environmental and sanitation management services.*

**Authors’ response:** We have done this (see revised manuscript, lines 133 – 134).

*31) Line 137-138 (Rephrase) This is largely due to restricted financial investments, along with limited operational resources and technical capacities.*

**Authors’ response:** As the Reviewer #2 suggested, we have done this (see revised manuscript, lines 137 - 139).

*32) Line 139-143 (Rephrase) The government’s response to outbreaks, led by the National Institute of Public Hygiene (NIPH) under the Ministry of Health (MoH) of Côte d’Ivoire, primarily relies on sporadic insecticide space spraying targeting adult Ae. mosquitoes, along with systematic removal, physical destruction, and/or treatment of larval breeding sites.*

**Authors’ response:** As the Reviewer #2 suggested, we rewrote the phrase according to his suggestions (see revised manuscript, lines 140 – 144).

*33) Line 152 delete the word “there”*

**Authors’ response:** As the Reviewer #2 suggested, we deleted « there » (see manuscript, line 153).

*34) Line 152-153 better to combine the two sentences in one.*

**Authors’ response:** As the Reviewer #2 suggested, we combined the two sentences in one sentence (see revised manuscript, line 153 – 154).

*35) Line 159 Ae.*

**Authors’ response:** As the Reviewer #2 suggested, we used *Ae*. instead *Aedes* (see revised manuscript, line 160).

*36) Line 180-186 (Rephrase) In this study, four urban municipalities were selected based on their epidemiological backgrounds and incidences of dengue (DEN) and yellow fever (YF): Bingerville (5° 21’ N; 3° 54’ W) and Cocody (5° 20’ N; 3° 58’ W), located in the Cocody-Bingerville health district where DEN and YF cases are regularly reported; Treichville (5° 19’ N; 4° 01’ W), situated in the Marcory-Treichville district with few recorded cases of DEN and YF; and Yopougon (5° 20’ N; 4° 00’ W), where DEN and YF cases are rare.*

**Authors’ response:** We thank the Reviewer #2 for giving this rephrase (see revised manuscript lines 182 – 188). However, as the Editor reported in the previous correction, «14) once 'dengue' has been abbreviated as 'DEN,' it should be consistently referred to as such for clarity and coherence in terminology usage.

*37) Line 188: reported between 2017 to 2024, according to hospital data.*

**Authors’ response:** We took into account the suggestion of the Reviewer #2 (see revised manuscript, line 190).

*38) Lines 210- 215 I think this sentence repeats the lines 180-188. Delete or rephrase.*

**Authors’ response:** We thank the Reviewer #2 for his suggestions. We have rephrased the sentences (see revised manuscript, lines 212 -217). We have described it as follows, to better explain the design of our study and show why we chose our study sites

*39) Line 222-223 don’t understand the meaning of this sentence.*

**Authors’ response:** We have rephrased the sentence for better understanding (see revised manuscript, line 224).

*40) Lines 329-330: check the percentages of each genus*

**Authors’ response:** We have checked the percentages of each genus and they are correct (see the overall section of table 1) and revised manuscript from line 337 to 338.

*41) Lines 329-330: I would suggest to present the number of each genus differently, it is not necessary to include the total number of mosquitoes each time, but you could present the number of males and females you collected for each genus and delete the ( before the word Aedes.*

**Authors’ response:** As the Reviewer #2 suggested, we took into account the suggestions (see revised manuscript, lines 337 – 338).

*42) Line 334 you have already share this information (line 329) about the proportion of Aedes genus*

**Authors’ response:** As the Reviewer #2 suggested, we took into account the suggestions (see revised manuscript, lines 345).

*43) Line 337 you have already share this information (lines 330-331)*

**Authors’ response:** As the Reviewer #2 suggested, we took into account the suggestions (see revised manuscript, lines 348).

*44) Lines 339-343 Other medically important non-Aedes species, such as Culex quinquefasciatus (2.65%, 2,298/86,796), a vector of arboviruses, and Anopheles gambiae s.l. (0.16%, 136/86,796), a vector of Plasmodium spp, were also collected across the four study areas, though in relatively low proportions.*

**Authors’ response:** As the Reviewer #2 suggested, we took into account the suggestions (see revised manuscript, lines 350 - 353).

*45) Lines 343-346 this information (the developmental stage of the collected mosquito) should be given from the beginning of the results section.*

**Authors’ response:** As suggested by the Reviewer #2, we have moved lines 343-346 from the end to the beginning of the results section (see revised manuscript, lines 339 – 342).

*46) Table 1: maybe delete the line with the subtotal, it is confusing.*

**Authors’ response:** We thank the reviewer for his suggestions. We set subtotal to give the proportion of each type of mosquito. In addition, the genera *Aedes* and *Culex* have more than one species in Gbagba and overall. This was done in order to standardise, even though there was only one species in some of the study sites.

**Reviewer#3**

**Summary and General** **Comments**

*47) Most of my previous comments were addressed but there is still some clarification to be done before accepting the manuscript for publication.*

**Authors’ response:** We would like to thank the Reviewer #3 for his encouragement and we will endeavor to consider any suggestions.

*48) Line 84. Ae. Aegypti*

**Authors’ response:** We corrected it according to reviewer #2 comments, he suggested since the first correction that *Ae. aegypti* should be written as the species name *Aedes aegypti* has already been written above. Also, we were reading and had seen *Ae. aegypti* written like that at the beginning of a sentence in the following manuscripts (**Dutra *et al*., 2024:** [**https://doi.org/10.1371/journal.pntd.0012482**](https://doi.org/10.1371/journal.pntd.0012482) **and Lopez-Rosero *et al*., 2024: https://doi.org/10.1371/journal.pntd.0011908**) (see revised manuscript line 85).

*49) Line 91. Aedes aegypti*

**Authors’ response:** We corrected it (see revised manuscript line 91).

*50) Line 94 outbreaks of DEN and YF*

**Authors’ response:** We took into account the suggestion of the Reviewer #3 (see revised manuscript, line 94).

*51) Line 121. “This Aedes species can transmit over 50 viruses to humans”. Please double check this.*

**Authors’ response:** As the Reviewer #3 suggested, we have checked and changed the bibliographic reference (see revised manuscript, lines 121 – 122). See reference number 10.

*52) Lines 175-176. “Abidjan is the first and the ninth largest city of Côte d’Ivoire and Africa, respectively”. Please add the reference*

**Authors’ response:** We took into account the suggestion of the Reviewer #3. So in the sentence we replaced « ninth » by «third in South Africa » (see revised manuscript, lines 176 - 177) and reference number 22.

*53) Lines 345-346. “The tendency of the dominance of Ae. aegypti (99.44%, 83,401/83,870) was observed mainly among the larval collections”. Please could you give the explanation?*

**Authors’ response:** We thank the Reviewer #3 for his suggestions. The sentence has been reworded to make it easier to understand (see revised manuscript, lines 341 – 342).

*54) Lines 583-587. “In general, the proportions of unfed females were higher during the rainy seasons (57.2 ± 4.1% in LRS and 52.9 ± 4.0% in SRS) compared with the dry seasons (42.0 ± 7.0% in LDS and 37.5 ± 7.0% in SDS) (S2 Fig). In contrast, the proportions of blood-fed females were higher during the dry seasons (30.0 ± 6.5% in LDS and 27.1 ± 6.4% in SDS) than during the rainy seasons (22.8 ± 3.5% in LRS and 17.6 ± 3.1% in SRS)”. This could be due to what? What is the potential implication in term of DEN or YF transmission? This result need to be discussed.*

**Authors’ response:** As suggested by the Reviewer #3, we discussed this result. (see revised manuscript, lines 737 – 743).

*55) Lines 596-598. “Aedes aegypti parity rate was higher in Ayakro (51.9 ± 4.9%, n = 106), followed by Gbagba (50.0 ± 4.9, n = 104), Entente (48.6 ± 4.9, n = 105), and Anono (48.1 ± 5.5%, n = 81)”. Since the difference is not statistically different, you cannot say parity was higher in a location.*

**Authors’ response:** We rewrote the sentence for a better understanding (see revised manuscript, lines 603 – 604).

*56) Line 604. “The parity rate was no significantly different”. Please replace no by not.*

**Authors’ response:** We replaced « no »by « not ». (see revised manuscript, line 611).

*57) Lines 648-650. A tentative explanation of why Ae. albopictus was not found is needed since it was showed that in several countries infested by Ae. albopictus few months later this species become a dominant species replacing the native species Ae. aegypti.*

**Authors’ response:** We gave an explanation as suggested by the Reviewer #3. (see revised manuscript, lines 660 – 669).

*58) Line 673-674. “The high Aedes-positivity of tires was due to the high presence of tires”. This explanation is too simplistic. Does make sense for me.*

**Authors’ response:** The sentence has been rewritten taking into account the sentence rewritten by reviewer #1 (see revised manuscript, line 693 – 694) or response 8).

*59) Line 701. These awareness campaigns were a part of public health responses coordinated by the NIPH of the MOH.  NIPH?  MOH?*

**Authors’ response:** We rewrote the sentence. However, NIPH means National Institute of Public Hygiene, and MHPH, Ministry of Health and Public Hygiene. We have abbreviated them here because they have already been defined above in the introduction section from lines 140 - 141. (See revised manuscript, lines 721 – 723).

*60) Line 724. Anthropophilicity?????*

**Authors’ response:** I thank the Reviewer #3 for bringing this to my attention. I corrected the word. (see revised manuscript, line 753).

*61) Lines 726-727. Where are the epidemiological data for DEN or YF?*

**Authors’ response:** This is not epidemiological data for dengue or yellow fever. We want to emphasize that Stegomyia indices alone do not necessarily confirm an arbovirus outbreak. Several factors must be present, such as the presence of infected mosquitoes and hosts carrying the viruses. However, dengue and yellow fever epidemics can occur even with lower value of these indices. See reference [30].

*62) Line 733. Please could you indicate an example of new tools?*

**Authors’ response:** We have provided some tools as suggested by the Reviewer #3. (see revised manuscript, lines 765 – 767).

*63) Lines 775-776. “The key larval breeding sites were water storage receptacles, tires and discarded containers mostly found outside of houses and during the rainy seasons”. Please could indicate how the key larval breeding sites were determined?*

**Authors’ response:** The main breeding sites of the *Ae. aegypti* vector were identified based on their positivity for the immature stages of *Aedes aegypti*, in contrast to other breeding sites that were less positive for larvae or pupae of *Ae. aegypti*.

**References**

*Please review your reference list to ensure that it is complete and correct. If you have cited papers that have been retracted, please include the rationale for doing so in the manuscript text, or remove these references and replace them with relevant current references. Any changes to the reference list should be mentioned in the rebuttal letter that accompanies your revised manuscript. If you need to cite a retracted article, indicate the article's retracted status in the References list and also include a citation and full reference for the retraction notice.*

**Authors’ response:** The bibliographical references have been updated to take account of certain references that have been added. Reference numbers are given in bold brackets: **References replaced:** **[10]**, line 122 and lines, 895 -897, **[30]**: lines 967 – 970, **[31]**: lines 971 – 972. **References added:** **[22]**: lines 942 – 945, **[37]**: lines 990 – 993, **[38]**: lines 994 – 998, **[40]**: lines 1005 – 1009, **[41]**: 1010 – 1013, **[42]**: lines 1015 – 1019, **[43]**: lines 1021 – 1023, **[58]**: lines 1085 – 1093, **[59]**: lines 1095 – 1097.
